# Knowledge, attitudes and practices of government animal health workers on antibiotic use and antibiotic resistance in Timor-Leste

**DOI:** 10.3389/fvets.2022.1063530

**Published:** 2022-11-24

**Authors:** Shawn Ting, Abrao Pereira, Amalia Alves, Paulo Gabriel Vong da Silva, Cristibela Dos Santos, Steven Davis, Hanna E. Sidjabat, Jennifer Yan, Joshua R. Francis, Joanita Bendita da Costa Jong, Tamsin S. Barnes

**Affiliations:** ^1^Global and Tropical Health Division, Menzies School of Health Research, Charles Darwin University, Darwin, NT, Australia; ^2^Ministry of Agriculture and Fisheries, Government of Timor-Leste, Dili, Timor-Leste; ^3^Epivet Pty. Ltd., Withcott, QLD, Australia; ^4^The University of Queensland, School of Veterinary Science, Gatton, QLD, Australia

**Keywords:** antibiotic use, knowledge, practices, antimicrobial resistance, Timor-Leste, animal health worker, antibiotics, technician

## Abstract

**Introduction:**

Antibiotic resistance is a global health threat, and there is growing concern on the inappropriate use of antibiotics in the livestock sector especially in low and middle income countries. The purpose of the study was to understand the knowledge, attitudes and practices on antibiotic use and antibiotic resistance of government animal health workers in Timor-Leste.

**Method:**

A cross-sectional survey using a census approach was conducted between August 2021 and January 2022 focusing on government animal health workers involved in field work and access to antibiotics. Interviews were face-to-face in the local Tetun language. Descriptive and regression analysis informed by causal diagrams were performed.

**Result:**

The study found poor knowledge of antibiotics among participants, with only 8.0% (13/162) able to correctly answer questions on how antibiotics worked. Knowledge of antibiotic resistance was poor as only 29.0% (47/162) of participants had heard of antibiotic resistance and were able to accurately identify that it made antibiotics less effective. Knowledge of antibiotics and knowledge of antibiotic resistance were crudely associated with being a veterinary technician and having university education. Attitude scores were positively influenced by knowledge of antibiotics and antibiotic resistance. Antibiotics were most commonly used in pigs, cattle and buffalo, with oxytetracycline being the most commonly used antibiotics in pigs and chicken. However, most participants reported a lack in supply of this antibiotic (137/162, 78.4%) and other antibiotics. Empiric use of antibiotics in sick animals was common, and some participants used antibiotics for parasitic diseases. Less than a fifth of participants reported ever using human antibiotics, and use of antibiotics for growth promotion was uncommon.

**Conclusion:**

There is a need to develop Timor-Leste specific treatment guidelines, strengthen veterinary diagnostic support, improve antibiotic procurement, and develop training programs to address knowledge gaps and poor practices found in this study.

## Introduction

Antibiotic resistance is a global health threat which has emerged rapidly in recent decades due to the inappropriate use of antibiotics in humans and animals (1, 2). In low and middle income countries (LMICs), the livestock sector has received significant attention due to concerns with a high level of antibiotic use for disease prevention and growth promotion (3–5). Furthermore, studies in such countries have shown inappropriate use of antibiotics by farmers and animal health professionals, and such behaviour is often accompanied by a poor understanding of antibiotics and antibiotic resistance (6–9). LMICs are also particularly susceptible to the impacts of antimicrobial resistance because of their under-resourced health systems (10, 11). These countries also have a lower capacity to address the problem of inappropriate antibiotic use because of inadequate policy, legislation and enforcement (10, 12).

Timor-Leste is a young LMIC (13) located in Southeast Asia on the east side of Timor island (14). The country has a population of about 1.3 million (15) and a land area of about 15,000 km^2^ (16). At the time the study was conducted, the country had 13 municipalities including Oecusse which is recognised as a Special Administrative Region that is physically separated from the rest of Timor-Leste by West Timor (16, 17). Each municipality is further divided into administrative posts followed by villages (16). The majority of the population in Timor-Leste live in rural communities, where many agriculture households rely on subsistence farming as their main livelihood (18). Based on the 2019 Agriculture Census, ownership of livestock is very high, where pigs, chickens and cattle are owned by 81.2, 77.6, and 40.8% of agriculture households respectively (19).

The Ministry of Agriculture and Fisheries (MAF) is responsible for providing veterinary and animal health services in Timor-Leste (19, 20). These responsibilities are divided across different levels of government. At the national level, the National Directorate of Veterinary coordinates animal health policies such as disease surveillance, prevention and control (21). At the municipal level, a Livestock and Veterinary Department focuses on the implementation of programmes and provides veterinary services directly to farmers (20). This includes providing free treatment services to animals owned by agriculture households, which can involve the administration of veterinary antibiotics (21). There are few private animal health services within the country (22), which includes <5 small private veterinary clinics focusing on companion animals that are based in the capital Dili (23). There is one veterinary laboratory in the country, which has capacity for bacteriology and antimicrobial susceptibility testing (24).

There is no local manufacture of veterinary antibiotics in Timor-Leste (25). All veterinary antibiotics are imported and applications to import antibiotics must be submitted to MAF for approval (25). MAF is the largest importer of veterinary antibiotics (25). These antibiotics are subsequently distributed to Livestock and Veterinary Departments in each municipality and are used by government animal health workers (GAHW) in different animals (26). There is no legislation or guidelines governing the prudent use of antibiotics in animals in the country (26). The use of veterinary antibiotics in Timor-Leste has been estimated to be lower than the global average after adjusting for animal biomass (25), and the most commonly used classes of veterinary antibiotics in animals are tetracycline's, penicillin's, and macrolides (25).

GAHWs in Timor-Leste include veterinarians, veterinary technicians, livestock technicians and extension workers. There are few qualified veterinarians in Timor-Leste (20) as is the case in several other developing countries (12, 27, 28), and most are based in the national office in administrative positions. Therefore, the responsibility of providing animal health services to farmers which includes the administration of antibiotics to animals lies mainly with veterinary technicians and livestock technicians (20), although some of these technicians are based at the national or municipal offices focusing on administrative roles (29).

Most veterinary technicians are animal health degree graduates from the national university where the first cohort graduated in 2013 (20). On the other hand, most livestock technicians are agriculture high school graduates with only some receiving university education (29). Many of the current livestock technicians were trained under an Agriculture Rehabilitation Project funded by World Bank around the time of Timor-Leste's restoration of independence in 2002 (30). Most government veterinary and livestock technicians are under the leadership of a municipal level Livestock and Veterinary Department, and are assigned to administrative post(s). Due to this arrangement, provision of animal health services may often be lacking in villages that are further away from the office of the administrative post. In such situations, extension workers employed by the National Directorate of Agriculture Extension who are present in almost every village (31) may be enlisted to provide treatment services including the administration of antibiotics to animals. However, the usual role of an extension worker involves improving agriculture productivity focusing mainly on crops and most have not received any formal animal health training (21, 32, 33). Additionally, volunteers who are usually students or recent graduates of the animal health degree from various universities may also participate in providing animal health services such as administering antibiotics to gain experience under the supervision of other GAHWs. In certain circumstances such as during a disease outbreak response or mass vaccination campaign, non-technical MAF employees may also be enlisted to provide assistance and may gain access to antibiotics.

In Timor-Leste, there have been no studies investigating the knowledge, attitudes and practices (KAP) of antibiotic use and antibiotic resistance among GAHWs, although there was a recent study which found very poor knowledge of antibiotics and antibiotic resistance among smallholder pig farmers (34). In other LMICs, studies investigating the KAP of antibiotic use among animal health workers has showed poor knowledge of antibiotic resistance and poor antibiotic use practices (9, 12, 35, 36). Some poor use practices identified in these studies include administering antibiotics based on farmer request, selecting antibiotics based on convenience rather than effectiveness, using antibiotics to treat non-bacterial infections, and using antibiotics as a substitute for poor biosecurity practices in disease prevention. Similarly, findings from a study investigating the KAP of antibiotic use and resistance in Timor-Leste could help identify knowledge gaps and any inappropriate use of antibiotics among GAHWs. This could then inform strategies to improve prudent use of antibiotics among GAHWs, which has potential to be impactful in combatting antibiotic resistance in animals since this group administers the majority of veterinary antibiotics in Timor-Leste (25). Furthermore, GAHWs are a common source of knowledge on antibiotic use for farmers in Timor-Leste (34), and are well-placed to promote and encourage prudent use among farmers provided they are well-trained. Therefore, this study aims to understand the knowledge, attitudes and practices on antibiotic use and antibiotic resistance of GAHWs in Timor-Leste.

## Materials and methods

### Study area, design, and sampling

A cross-sectional study was conducted among GAHWs between August 2021 and January 2022 in all 13 municipalities of Timor-Leste. The study was undertaken as part of a larger Fleming Fund Country Grant project administered by the Menzies School of Health Research (Menzies) which aims to optimise antimicrobial use across both human and animal health sectors (37). Eligibility criteria for inclusion in the study were:

1. Veterinary technicians and livestock technicians who were involved in field work. Field work means being in a role that involves being called out to provide animal health services such as vaccination and treatment on a regular basis.
2. Extension workers, other MAF employees or volunteers with access to antibiotics for work. Access to antibiotics means that antibiotics distributed to the municipal level Livestock and Veterinary Department office are provided to these individuals for use in animals.

The size of the target population was estimated to be about 170 based on information obtained from the MAF national office. A census approach was considered logistically feasible and was implemented. Before each municipality was visited, the municipal-level Livestock and Veterinary Department office was contacted by Menzies to provide an exact list of individuals which met the eligibility criteria because information on the daily roles and responsibilities of each staff which informed inclusion or exclusion from the study was not available at the national level. All eligible participants were contacted and invited to participate in the interview 1–2 weeks before the scheduled interview date. Participants that did not live close to the interview venue in each municipality were provided with monetary compensation for their travel expenses.

### Data collection

The questionnaire was initially developed in English and translated into Tetun by two authors (A.P, A.A) who are fluent in both languages. The questionnaire was piloted with five non-government veterinary technicians and refined prior to implementation. The final questionnaire had four sections and 122 questions which were mostly closed-ended, and an English version is available in the Supplementary material.

The first section focused on the participant's demographic information, use of laboratory services, and observations on farm properties they have visited. The second section assessed the knowledge of GAHWs on antibiotics, antibiotic resistance and antibiotic residues. The third section assessed the attitudes of GAHWs towards antibiotic use, which can be defined as their beliefs about inappropriate antibiotic use and reducing antibiotic use through measures such as vaccination, farm biosecurity and good animal husbandry. The final section collected information on the antibiotic use practices in animals.

The interview was conducted face-to-face in the Tetun language by two trained Timorese animal health professionals and took an average of 50 min to complete. Picture aids were used when asking participants about specific antibiotic products to aid recollection. To minimise interview fatigue, the interview was divided into 2 sessions with a short break in between, and both interviewers were consistently assigned to ask the same set of questions throughout the entire study. Almost all the interviews were conducted either in the MAF national or municipal office, except for 6 GAHWs from Dili municipality who were interviewed in the Menzies Office in Dili to allow better flexibility on the interview time.

All responses were collected using the REDCap Mobile App on Android tablets as this facilitated offline data collection due to limited internet connectivity in some municipalities. Any additional responses provided by a participant which provided Supplementary information was captured in free-text format. Subsequently, responses were uploaded and managed using REDcap electronic data capture tools hosted at Menzies (38, 39). All responses were checked for data entry errors by two members of the research team.

### Data management

Free-text responses to 14 questions that allowed open-ended responses were classified into categories that were developed retrospectively by five members of the research team. An overall attitude score was created for each participant by assigning a score of 1 to each of the 11 attitude questions that were answered correctly. Binary variables were created for several of the demographic attributes for use in regression analyses: experience was categorised as <10 years/≥10 years, highest education as university/not university and learnt about antibiotics at a training course as yes (MAF or non-MAF training)/no.

### Data analysis

Categorical variables were described using absolute and relative frequency. Continuous variables were summarised using median and interquartile range (IQR). Causal diagram-informed analyses were proposed *a priori* to explore relationships between several sets of variables: (1) demographic variables and knowledge of how antibiotics work (i.e. identifying that antibiotics kill bacteria but not viruses), (2) demographic variables and knowledge of antibiotic resistance (i.e. correctly identifying that antibiotic resistance reduces the effectiveness of antibiotics) and (3) key knowledge variables and attitude score. A causal diagram was developed for each of the outcome variables of interest. Prior to conducting the analyses, relationships between pairs of explanatory variables were assessed through correlations and cross-tabulations and planned analyses adapted as required. Where appropriate, the causal diagrams were used to inform minimal sufficient adjustment sets of covariates to estimate total and direct effects for each variable of interest. Logistic regression analyses were conducted for binary outcome variables and linear regression analyses for attitude score. The distributions of residuals from all linear regression models were checked for normality. All analyses included municipality as a random effect to account for clustering at this level. All data analyses were conducted using Stata 17.0 (40).

## Results

### Participation

**Figure 1:**
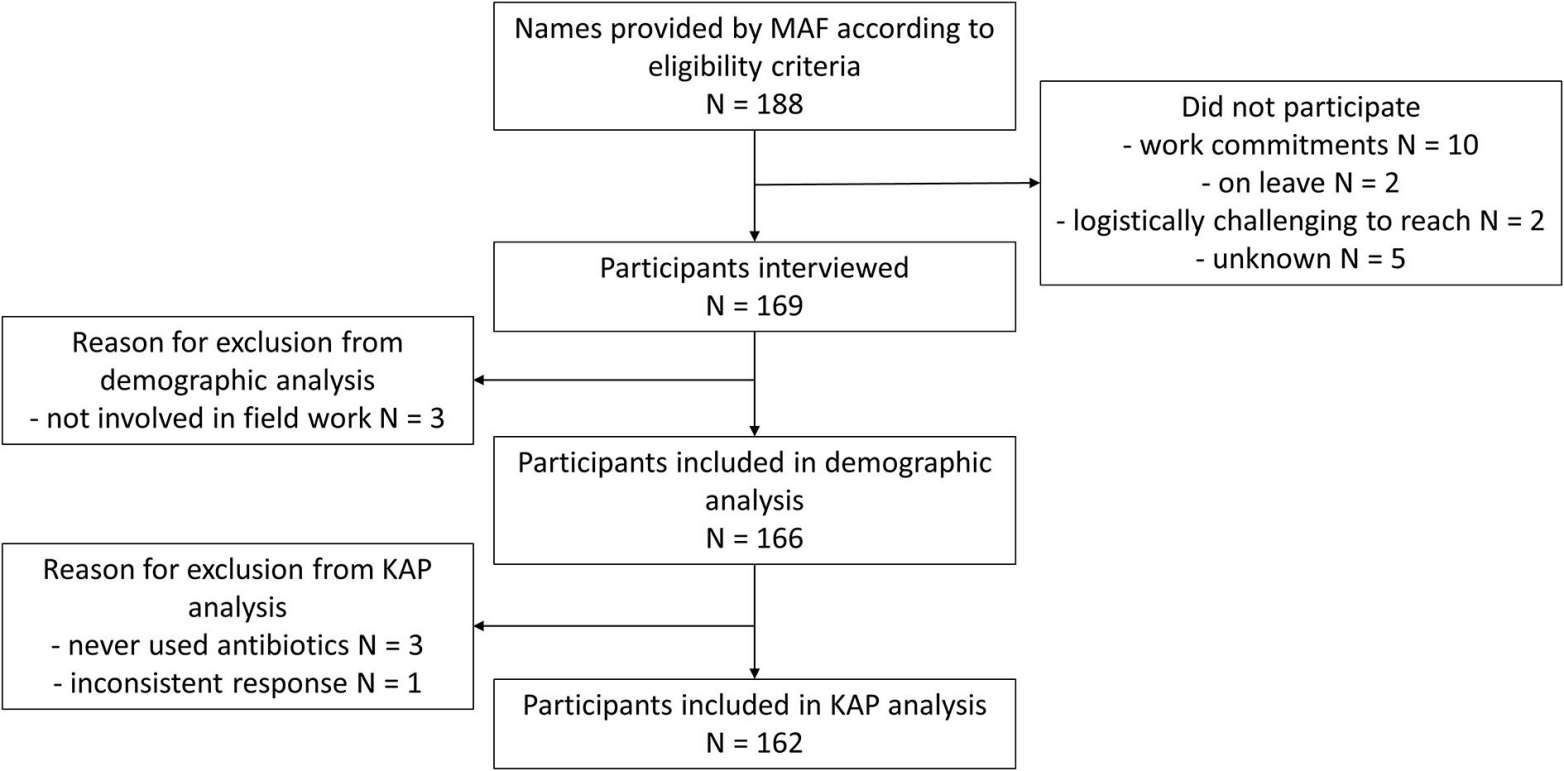
Study flowchart providing reasons for non-participation and exclusion from analysis.

A total of 188 names were provided to Menzies by the various municipal-level Livestock and Veterinary Department offices. Of these, 169 were interviewed giving an overall participation rate of 89.9%. The most common reasons for being unable to participate was having other work commitments. After the interviews were conducted, we identified three participants who did not meet the eligibility criteria and these participants were removed the dataset. Separately, we identified three participants who had met the eligibility criteria, but had never used any antibiotics for work mainly because they had just joined the government animal health workforce and one participant who gave inconsistent responses throughout the interview. These four participants were included in the demographic analysis but excluded from subsequent analyses. Therefore, 166 participants were included in the demographic analyses and 162 participants were included in the knowledge, attitudes and practices analyses. A study flowchart can be found in Figure 1.

### Participant demographics

**Figure 2:**
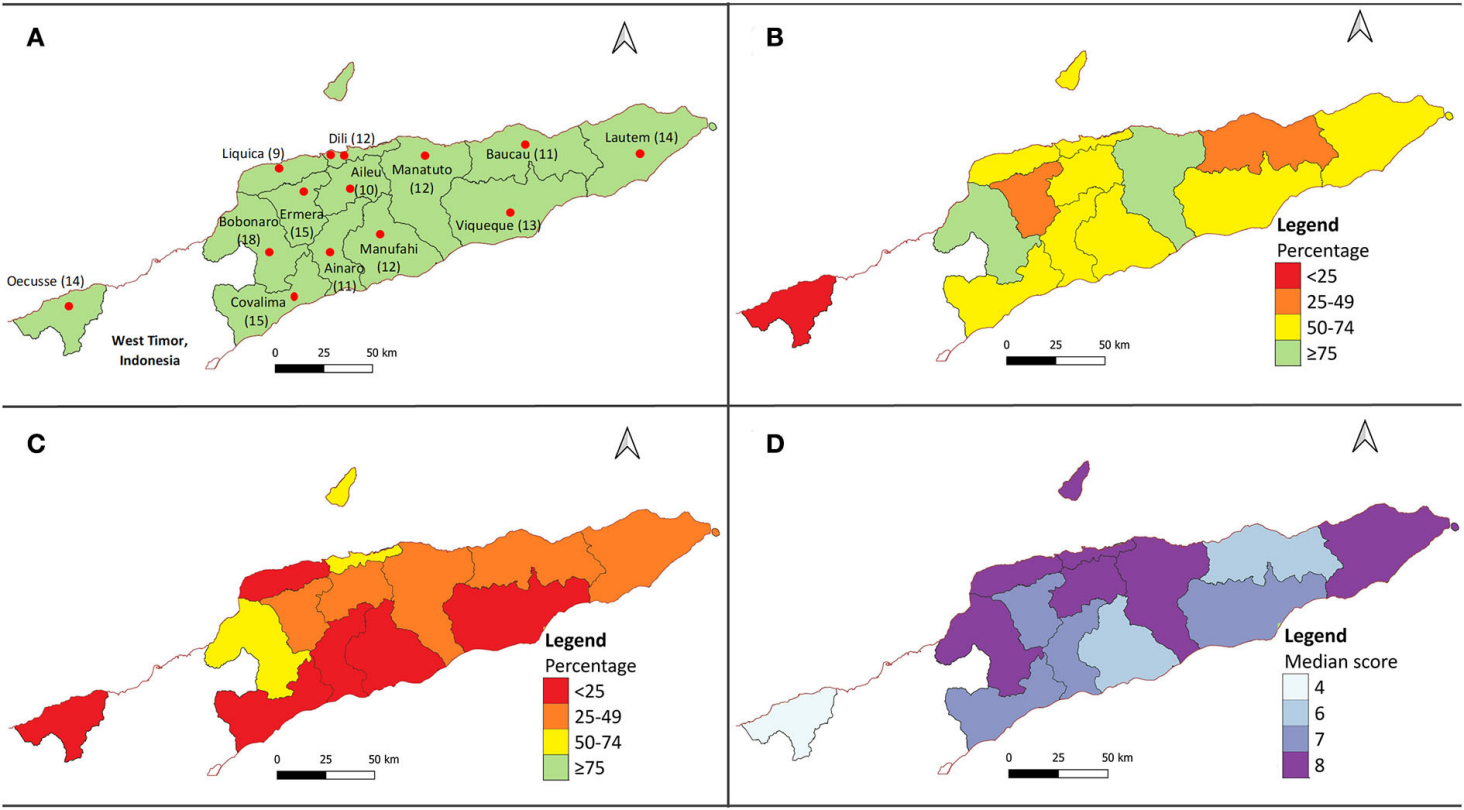
Map of Timor-Leste showing **(A)** all 13 municipalities where numbers shown after the name of each municipality refer to the number of government animal health workers interviewed in each location. The red circles represent the locations where interviews were conducted in each municipality. **(B)** Percentage of participants who had knowledge of how antibiotics worked. **(C)** Percentage of participants who had knowledge of antibiotic resistance. **(D)** Median attitude score.

Participant demographics are summarised in Table 1, with further information available in Supplementary Tables 1, 2. The number of participants and location of interview site(s) in each municipality is shown in Figure 2A. Of 166 participants, 87.3% (145/166) were male and 12.7% (21/166) were female. The most common age group among participants was 30−39 years old. About one-third of participants (57/166, 34.3%) were veterinary technicians, slightly more than half (87/166, 52.4%) were livestock technicians and just over one-tenth (22/166, 13.3%) were classified as others which includes extension workers, volunteers or non-technical MAF employees. Almost all veterinary technicians (53/57, 93.0%) had a university degree in animal health and most livestock technicians (53/87, 60.9%) completed senior high school focusing on agriculture without a university education. Participants had a median of 9 (IQR 8−11) years of experience performing field work. The median number of farms visited per month for the last 12 months for veterinary technicians (median = 15; IQR 7.5−23.5) and livestock technicians (median = 15; IQR 8−20) was higher than those classified as others (median = 5; IQR 3.75−10). Almost all participants stated that vaccination (159/166, 95.8%) or treatment (165/166, 99.4%) were their main reasons for visiting farms between a 12-month period from February 2020 to February 2021. Almost all participants (164/166, 98.8%) knew that samples from sick animals can be sent to laboratory for diagnosis. 70.5% (117/166) have ever sent samples from sick animals to the laboratory for diagnosis, with the majority (85/117, 72.6%) only sending 1 to 5 samples in the last 12 months. Of those that have ever sent samples from sick animals to the laboratory, the majority (82/117, 70.1%) said they have never received a laboratory result.

**Table 1:** Demographic information of 166 government animal health workers (overall and by position) surveyed in Timor-Leste.

**Attribute**	**Veterinary technicians (*n =* 57)**	**Livestock technicians (*n =* 87)**	**Other positions (*n =* 22)**	**Overall (*n =* 166)**
**Gender**				
Male	40 (70.2)	85 (97.7)	20 (90.9)	145 (87.3)
Female	17 (29.8)	2 (2.3)	2 (9.1)	21 (12.7)
**Age (years)**				
<30	10 (17.5)	0 (0.0)	3 (13.6)	13 (7.8)
30–39	43 (75.4)	16 (18.4)	5 (22.7)	64 (38.6)
40–49	2 (3.5)	40 (46.0)	8 (36.4)	50 (30.1)
≥ 50	2 (3.5)	31 (35.6)	6 (27.3)	39 (23.5)
**Highest education**				
University—animal science	2 (3.5)	13 (14.9)	2 (9.1)	17 (10.2)
University—animal health	53 (93.0)	0 (0.0)	1 (4.5)	54 (32.5)
University—other	0 (0.0)	4 (4.6)	7 (31.8)	11 (6.6)
Senior high school—agriculture	2 (3.5)	53 (60.9)	11 (50.0)	66 (39.8)
Senior high school—other	0 (0.0)	13 (14.9)	1 (4.5)	14 (8.4)
Junior high school	0 (0.0)	3 (3.4)	0 (0.0)	3 (1.8)
Others	0 (0.0)	1 (1.1)	0 (0.0)	1 (0.6)
**Work location**				
Home	2 (3.5)	11 (12.6)	13 (59.1)	26 (15.7)
Municipal office	26 (45.6)	28 (32.2)	5 (22.7)	59 (35.5)
Posto Administrativo office	12 (21.1)	28 (32.2)	2 (9.1)	42 (25.3)
Animal Health Centre	11 (19.3)	8 (9.2)	1 (4.5)	20 (12.0)
National Directorate of Veterinary	5 (8.8)	2 (2.3)	0 (0.0)	7 (4.2)
Animal Production Centre	0 (0.0)	6 (6.9)	0 (0.0)	6 (3.6)
Extension Centre	1 (1.8)	4 (4.6)	1 (4.5)	6 (3.6)
**Purpose of farm visit** ^a^				
Vaccination	56 (98.2)	84 (96.6)	19 (86.4)	159 (95.8)
Treatment of sick animals	57 (100.0)	86 (98.9)	22 (100.0)	165 (99.4)
Advice on livestock disease	9 (15.8)	11 (12.6)	2 (9.1)	22 (13.3)
Advice on livestock management	9 (15.8)	17 (19.5)	1 (4.5)	27 (16.3)
Field project implementation	7 (12.3)	10 (11.5)	0 (0.0)	17 (10.2)
Animal health data collection	1 (1.8)	2 (2.3)	2 (9.1)	5 (3.0)
Other	5 (8.8)	2 (2.3)	0 (0.0)	7 (4.2)
**Know send samples to laboratory**				
Yes	57 (100.0)	86 (98.9)	21 (95.5)	164 (98.8)
No	0 (0.0)	1 (1.1)	1 (4.5)	2 (1.2)
**Ever sent samples to laboratory**				
Yes	43 (75.4)	65 (74.7)	9 (40.9)	117 (70.5)
No	14 (24.6)	21 (24.1)	12 (54.5)	47 (28.3)
**Number of samples from sick animals sent to laboratory in last 12 months** ^b^				
1–5	27 (62.8)	49 (75.4)	9 (100.0)	85 (72.6)
6–10	2 (4.7)	5 (7.7)	0 (0.0)	7 (6.0)
11–20	2 (4.7)	3 (4.6)	0 (0.0)	5 (4.3)
> 20	11 (25.6)	7 (10.8)	0 (0.0)	18 (15.4)
Not stated	1 (2.3)	1 (1.5)	0 (0.0)	2 (1.7)
**How often lab test result received** ^**b**^				
Always	3 (7.0)	2 (3.1)	0 (0.0)	5 (4.3)
Sometimes	15 (34.9)	13 (20.0)	2 (22.2)	30 (25.6)
Never	25 (58.1)	50 (76.9)	7 (77.8)	82 (70.1)

a Multiple responses allowed.

b Of those who have previously sent samples to the laboratory (n = 117).

### Farm observations

At the time of the survey participants generally thought that current vaccination coverage in the areas they covered for work was low with only 24.7% (41/166) of participants thinking at least 67% chickens were vaccinated for Newcastle Disease and 4.8% (8/166) thinking that at least 67% pigs were vaccinated for Classical Swine Fever. More than half of the participants said that they occasionally observed fencing around farms with chickens and pigs (107/166, 64.5%), chickens in cages (132/166, 79.5%) and animals in pens (105/166, 63.3%). More than half had never observed a locked gate on farms with chickens and pigs (89/166, 53.6%), and more than three-quarters of participants had never observed dedicated boots (126/166, 75.9%), disinfection of footwear (133/166, 80.1%) or visitor sign-in books (136/166, 81.9%) on farms that they have visited. Based on comments captured from participants, farms that had higher biosecurity standards such as dedicated books and visitor sign often belonged to agriculture schools, religious organisations, commercial enterprises, or farmer groups supported by the government or aid programmes. Further information on farm observations can be found in Supplementary Table 3.

### Knowledge of antibiotics and antibiotic resistance

#### Knowledge of antibiotics

Knowledge of antibiotics by position can be found in Table 2, and further information found in Supplementary Table 4. All of the participants said they knew what antibiotics were, and the majority (107/162, 66.0%) said that one of the sources of this knowledge was a MAF training course. Most participants (142/162, 87.7%) correctly identified that antibiotics killed or inhibited bacteria. However, only slightly more than half of the participants correctly identified that antibiotics did not kill or inhibit virus (97/162, 59.9%). Less than 10% of the participants correctly identified that antibiotics did not directly reduce inflammation (11/162, 6.8%) or fever (16/162, 9.9%). Only nine veterinary technicians and four livestock technicians were able to answer all four questions on antibiotics correctly.

**Table 2:** Knowledge of antibiotics (overall and by position) of government animal health workers.

**Attribute**	**Veterinary technicians (*n =* 56)**	**Livestock technicians (*n =* 85)**	**Other positions (*n =* 21)**	**Overall (*n =* 162)**
**Know what antibiotics are**				
Yes	56 (100.0)	85 (100.0)	21 (100.0)	162 (100.0)
No	0 (0.0)	0 (0.0)	0 (0.0)	0 (0.0)
**Where learnt about antibiotics**				
University	47 (83.9)	10 (11.8)	3 (14.3)	60 (37.0)
Senior high school	7 (12.5)	36 (42.4)	7 (33.3)	50 (30.9)
At work	5 (8.9)	10 (11.8)	4 (19.0)	19 (11.7)
MAF training course	23 (41.1)	68 (80.0)	16 (76.2)	107 (66.0)
Non-MAF training course	2 (3.6)	1 (1.2)	0 (0.0)	3 (1.9)
Other	0 (0.0)	1 (1.2)	0 (0.0)	1 (0.6)
**Antibiotic kills bacteria**				
Correct	49 (87.5)	76 (89.4)	17 (81.0)	142 (87.7)
Incorrect	5 (8.9)	4 (4.7)	2 (9.5)	11 (6.8)
Don't know	2 (3.6)	5 (5.9)	2 (9.5)	9 (5.6)
**Antibiotic kills virus**				
Correct	43 (76.8)	46 (54.1)	8 (38.1)	97 (59.9)
Incorrect	11 (19.6)	31 (36.5)	11 (52.4)	53 (32.7)
Don't know	2 (3.6)	8 (9.4)	2 (9.5)	12 (7.4)
**Antibiotic directly reduces inflammation**				
Correct	8 (14.3)	3 (3.5)	0 (0.0)	11 (6.8)
Incorrect	46 (82.1)	76 (89.4)	19 (90.5)	141 (87.0)
Don't know	2 (3.6)	6 (7.1)	2 (9.5)	10 (6.2)
**Antibiotic directly reduces fever**				
Correct	10 (17.9)	6 (7.1)	0 (0.0)	16 (9.9)
Incorrect	45 (80.4)	76 (89.4)	19 (90.5)	140 (86.4)
Don't know	1 (1.8)	3 (3.5)	2 (9.5)	6 (3.7)
**Answered four questions on antibiotics correctly**				
	9 (16.1)	4 (4.7)	0 (0.0)	13 (8.0)
**Answered the effect of antibiotics on bacteria and virus correctly**				
	40 (71.4)	45 (52.9)	8 (38.1)	93 (57.4)
**Identified Medoxy-LA as an antibiotic**				
Correct	55 (98.2)	84 (98.8)	19 (90.5)	158 (97.5)
Incorrect	1 (1.8)	1 (1.2)	2 (9.5)	4 (2.5)
Don't know	0 (0.0)	0 (0.0)	0 (0.0)	0 (0.0)
**Heard about broad and narrow spectrum antibiotics**				
Yes	48 (85.7)	54 (63.5)	11 (52.4)	113 (69.8)
No	8 (14.3)	31 (36.5)	10 (47.6)	49 (30.2)
**Identified Medoxy-LA as broad spectrum antibiotic** ^a^				
Correct	43 (89.6)	47 (87.0)	6 (54.5)	96 (85.0)
Incorrect	1 (2.1)	4 (7.4)	3 (27.3)	8 (7.1)
Don't know	4 (8.3)	3 (5.6)	2 (18.2)	9 (8.0)
**Heard of critically important antimicrobial for human medicine**				
Yes	21 (37.5)	32 (37.6)	11 (52.4)	64 (39.5)
No	35 (62.5)	53 (62.4)	10 (47.6)	98 (60.5)

a Of those who had heard of broad and narrow spectrum antibiotics (n = 113).

Almost all participants (158/162, 97.5%) were able to correctly identify Medoxy-LA which is a long-acting oxytetracycline injectable and the most commonly used veterinary antibiotic in Timor-Leste (25). However, close to half of the participants (67/162, 41.4%) incorrectly identified ivermectin as an antibiotic. The majority of participants (113/162, 69.8%) had heard of broad and narrow spectrum antibiotics, and most of these participants (96/113, 85.0%) were able to able to correctly identify Medoxy-LA as a broad spectrum antibiotic. Less than half (64/162, 39.5%) had heard of critically important antimicrobial for human medicine. Most participants (145/162, 89.5%) had heard about the need to wait a few days after giving antibiotics before slaughtering or selling animals, and around three-quarter of participants (122/162, 75.3%) had heard about the risk of antibiotic residues after antibiotics are used in animals.

#### Knowledge of antibiotic resistance

Knowledge of antibiotic resistance by position is shown in Table 3. More than half of the participants (97/162, 59.9%) had heard of antibiotic resistance. Of those that have heard of antibiotic resistance, 78.4% (76/97) said they knew what antibiotic resistance was, and the majority (41/76, 53.9%) said that one of the sources of this knowledge was a MAF training course. Of those who said they knew what antibiotic resistance was, 61.8% (47/76) were able to correctly identify that resistance made antibiotics less effective. After it was clarified to all participants that antibiotic resistance made antibiotics less effective, 84.6% (137/162) and 77.2% (125/162) thought that it was a serious animal and human health issue respectively. Most participants (117/162, 72.2%) said that antibiotic resistance in animals can lead to antibiotic resistance in humans. Less than a third of participants had heard of either World Antimicrobial Awareness Week or Timor-Leste's National Action Plan for Antimicrobial Resistance.

**Table 3:** Knowledge of antibiotic resistance (overall and by position) of government animal health workers.

**Attribute**	**Veterinary technicians (*n =* 56)**	**Livestock technicians (*n =* 85)**	**Other positions (*n =* 21)**	**Overall (*n =* 162)**
**Heard of antibiotic resistance**				
Yes	43 (76.8)	43 (50.6)	11 (52.4)	97 (59.9)
No	13 (23.2)	42 (49.4)	10 (47.6)	65 (40.1)
**Think know what antibiotic resistance is** ^a^				
Yes	37 (86.0)	31 (72.1)	8 (72.7)	76 (78.4)
No	6 (14.0)	12 (27.9)	3 (27.3)	21 (21.6)
**Where learnt about antibiotic resistance** ^b^				
University	17 (45.9)	6 (19.4)	0 (0.0)	23 (30.3)
Senior high school	2 (5.4)	11 (35.5)	0 (0.0)	13 (17.1)
At work	7 (18.9)	6 (19.4)	2 (25.0)	15 (19.7)
MAF training course	12 (32.4)	23 (74.2)	6 (75.0)	41 (53.9)
Non-MAF training course	6 (16.2)	5 (16.1)	0 (0.0)	11 (14.5)
Media	3 (8.1)	0 (0.0)	1 (12.5)	4 (5.3)
Self-learning	3 (8.1)	0 (0.0)	0 (0.0)	3 (3.9)
**Effect of antibiotic resistance** ^b^				
Less effective	25 (67.6)	16 (51.6)	6 (75.0)	47 (61.8)
More effective	6 (16.2)	8 (25.8)	1 (12.5)	15 (19.7)
No change	2 (5.4)	4 (12.9)	0 (0.0)	6 (7.9)
Don't know	4 (10.8)	3 (9.7)	1 (12.5)	8 (10.5)
**Think antibiotic resistance is a serious animal health issue**				
Yes	51 (91.1)	66 (77.6)	20 (95.2)	137 (84.6)
No	3 (5.4)	9 (10.6)	0 (0.0)	12 (7.4)
Don't know	2 (3.6)	10 (11.8)	1 (4.8)	13 (8.0)
**Think antibiotic resistance is a serious human health issue**				
Yes	46 (82.1)	60 (70.6)	19 (90.5)	125 (77.2)
No	2 (3.6)	9 (10.6)	1 (4.8)	12 (7.4)
Don't know	8 (14.3)	16 (18.8)	1 (4.8)	25 (15.4)
**Think resistance in animals can lead to resistance in humans**				
Yes	43 (76.8)	57 (67.1)	17 (81.0)	117 (72.2)
No	5 (8.9)	11 (12.9)	1 (4.8)	17 (10.5)
Don't know	8 (14.3)	17 (20.0)	3 (14.3)	28 (17.3)
**Heard of world antimicrobial awareness week**				
Yes	13 (23.2)	19 (22.4)	3 (14.3)	35 (21.6)
No	43 (76.8)	66 (77.6)	18 (85.7)	127 (78.4)
**Heard of national action plan for antimicrobial resistance for timor-leste**				
Yes	21 (37.5)	19 (22.4)	5 (23.8)	45 (27.8)
No	35 (62.5)	66 (77.6)	16 (76.2)	117 (72.2)

a Of those who had heard of antibiotic resistance (n = 97).

b Of those who thought they knew what antibiotic resistance is (n = 76).

Bobonaro municipality had the highest proportion of GAHWs that had knowledge of how antibiotics worked and knowledge of antibiotic resistance. Oecusse and Ainaro municipalities had the lowest proportion of GAHW that had knowledge of how antibiotics worked and knowledge of antibiotic resistance respectively. The percentages of participants in each municipality who had knowledge of how antibiotics work (i.e. identifying that antibiotics kill bacteria but not viruses) and knowledge of antibiotic resistance are shown in Figures 2B,C respectively.

### Attitudes

The overall median score for attitudes on antibiotic use among participants was 7 out of 11 (IQR 6−8). Veterinary technicians had a higher median score (median score = 8) compared to the other participants (median score = 7). Participants from Oecusse municipality had the lowest median score (median score = 4), and median attitude scores by municipality are shown in Figure 2D. More than three-quarters of participants (127/162, 78.4%) believed that giving a broad spectrum antibiotic was always a better choice than a narrow spectrum antibiotic. Around two-third of participants (108/162, 66.7%) believed that it was appropriate to give an antibiotic for a shorter duration than recommended on the drug label if a sick animal is recovering. About one-fifth of participants (32/162, 19.8%) believed in giving healthy animals antibiotics to promote growth. Further information on responses to attitudes towards antibiotic use can be found in Supplementary Table 5.

### Antibiotic use practices

All 162 participants included in analysis reported ever giving antibiotics to animals for work, with most participants stating that they treated at least an average of 11 animals per month in the last 12 months. The key antibiotic use practices by position are detailed in Table 4.

**Table 4:** Antibiotic use practices (overall and by position) of government animal health workers.

**Attribute**	**Veterinary technicians (*n =* 56)**	**Livestock technicians (*n =* 85)**	**Other positions (*n =* 21)**	**Overall (*n =* 162)**
**Ever used antibiotics for work**				
Yes	56 (100.0)	85 (100.0)	21 (100.0)	162 (100.0)
No	0 (0.0)	0 (0.0)	0 (0.0)	0 (0.0)
Don't know	0 (0.0)	0 (0.0)	0 (0.0)	0 (0.0)
**Average number of animals treated with antibiotics per month prior to COVID-19**				
0–5	10 (17.9)	13 (15.3)	9 (42.9)	32 (19.8)
6–10	12 (21.4)	20 (23.5)	7 (33.3)	39 (24.1)
11–15	8 (14.3)	12 (14.1)	1 (4.8)	21 (13.0)
16–20	11 (19.6)	20 (23.5)	2 (9.5)	33 (20.4)
> 20	15 (26.8)	20 (23.5)	2 (9.5)	37 (22.8)
**Used antibiotics in sick animals to help them recover**				
Yes	56 (100.0)	85 (100.0)	20 (95.2)	161 (99.4)
No	0 (0.0)	0 (0.0)	0 (0.0)	0 (0.0)
Don't know	0 (0.0)	0 (0.0)	1 (4.8)	1 (0.6)
**Used antibiotics in healthy animals in contact with sick animals**				
Yes	26 (46.4)	45 (52.9)	9 (42.9)	80 (49.4)
No	30 (53.6)	40 (47.1)	12 (57.1)	82 (50.6)
**Used antibiotics to help healthy animals grow faster**				
Yes	1 (1.8)	5 (5.9)	0 (0.0)	6 (3.7)
No	55 (98.2)	80 (94.1)	21 (100.0)	156 (96.3)
**Always give antibiotics to sick animals**				
Yes	48 (85.7)	67 (78.8)	18 (85.7)	133 (82.1)
No	8 (14.3)	18 (21.2)	3 (14.3)	29 (17.9)
**Ever obtained antibiotics for work from sources other than MAF**				
Yes	12 (21.4)	23 (27.1)	1 (4.8)	36 (22.2)
No	44 (78.6)	62 (72.9)	20 (95.2)	126 (77.8)
**Top 3 species in which you used antibiotics in last 12 months**				
Local chicken	10 (17.9)	6 (7.1)	1 (4.8)	17 (10.5)
Broiler	0 (0.0)	0 (0.0)	0 (0.0)	0 (0.0)
Layer	0 (0.0)	1 (1.2)	0 (0.0)	1 (0.6)
Fighting cock	3 (5.4)	0 (0.0)	0 (0.0)	3 (1.9)
Pig	50 (89.3)	70 (82.4)	18 (85.7)	138 (85.2)
Cattle	42 (75.0)	76 (89.4)	19 (90.5)	137 (84.6)
Buffalo	12 (21.4)	52 (61.2)	9 (42.9)	73 (45.1)
Goat	27 (48.2)	35 (41.2)	10 (47.6)	72 (44.4)
Dog	23 (41.1)	13 (15.3)	6 (28.6)	42 (25.9)
Cat	0 (0.0)	0 (0.0)	0 (0.0)	0 (0.0)
Horses	1 (1.8)	3 (3.5)	0 (0.0)	4 (2.5)
None of the above	0 (0.0)	0 (0.0)	0 (0.0)	0 (0.0)
**Given human antibiotics to animals**				
Yes	9 (16.1)	11 (12.9)	5 (23.8)	25 (15.4)
No	47 (83.9)	74 (87.1)	16 (76.2)	137 (84.6)
**Given antibiotics though water to animals**				
Yes	9 (16.1)	3 (3.5)	3 (14.3)	15 (9.3)
No	47 (83.9)	82 (96.5)	18 (85.7)	147 (90.7)
Don't know	0 (0.0)	0 (0.0)	0 (0.0)	0 (0.0)
**Cheque the expiry date of antibiotics before using**				
Always	44 (78.6)	69 (81.2)	19 (90.5)	132 (81.5)
Most of the time	7 (12.5)	8 (9.4)	0 (0.0)	15 (9.3)
Sometimes	5 (8.9)	7 (8.2)	1 (4.8)	13 (8.0)
Never	0 (0.0)	1 (1.2)	1 (4.8)	2 (1.2)
**Ever given a lower antibiotic dose than on the label**				
Yes	27 (48.2)	22 (25.9)	3 (14.3)	52 (32.1)
No	28 (50.0)	61 (71.8)	18 (85.7)	107 (66.0)
Don't know	1 (1.8)	2 (2.4)	0 (0.0)	3 (1.9)
**Ever given a shorter duration of antibiotic treatment than on the label**				
Yes	22 (39.3)	20 (23.5)	6 (28.6)	48 (29.6)
No	34 (60.7)	63 (74.1)	15 (71.4)	112 (69.1)
Don't know	0 (0.0)	2 (2.4)	0 (0.0)	2 (1.2)
**Advise farmers to wait for a few days after giving antibiotics before slaughter/sale/eating**				
Always	47 (83.9)	59 (69.4)	17 (81.0)	123 (75.9)
Most of the time	5 (8.9)	10 (11.8)	1 (4.8)	16 (9.9)
Sometimes	2 (3.6)	12 (14.1)	0 (0.0)	14 (8.6)
Never	2 (3.6)	4 (4.7)	3 (14.3)	9 (5.6)
**Ever given farmers antibiotics to inject animals themselves**				
Yes	22 (39.3)	13 (15.3)	3 (14.3)	38 (23.5)
No	34 (60.7)	72 (84.7)	18 (85.7)	124 (76.5)
**Ever advised farmers to give antibiotics without first examining the animals**				
Yes	2 (3.6)	1 (1.2)	0 (0.0)	3 (1.9)
No	54 (96.4)	84 (98.8)	21 (100.0)	159 (98.1)

#### Reason for antibiotic use

Almost all participants (161/162, 99.4%) reported ever using antibiotics for treatment of sick animals and slightly less than half (80/162, 49.4%) reported ever using antibiotics for disease prevention. Very few participants have ever used antibiotics for growth promotion (6/162, 3.7%). In sick animals, the common clinical signs prompting use of antibiotics were diarrhoea (*n* = 88), fever (*n* = 59), cough (*n* = 53), lethargy (*n* = 53) swelling in body parts (*n* = 51), inappetence (*n* = 46) and wounds (*n* = 46). Some participants also used antibiotics for scabies (*n* = 20) and gastrointestinal worms (*n* = 1). Less than one-fifth of participants (29/162, 17.9%) did not always give antibiotics to sick animals, and the main reasons were unlikeliness of a bacterial infection or unavailability of antibiotics. Only seven out of these 29 participants reported facing resistance from farmers when they decided not to use antibiotics. Further information on reasons for antibiotic use can be found in Supplementary Table 6.

#### Antibiotic supply and choice

Most participants said that veterinary medicines including commonly used antibiotics such as Medoxy-LA (137/162, 84.6%) and Pen-Strep (112/162, 69.1%) were lacking in supply for work in the last 12 months. Almost a quarter (36/162, 22.2%) of participants said that they have ever obtained antibiotics from other sources other than MAF for work purposes, and almost all of them obtained antibiotics from agriculture shops (*n* = 32).

When deciding on which antibiotics to use, the majority of participants said that they always chose an antibiotic based on past experience (152/162, 93.8%), what the drug label states is effective for the suspected disease (151/162, 93.2%), drug availability (145/162, 89.5%), what was learnt at a training course (124/162, 76.5%), what was learnt at university/high school (121/162, 74.7%) and the duration of action (120/162, 74.1%). Almost all participants said that they never chose an antibiotic based on laboratory test results (153/162, 94.4%) and veterinary antibiotic prescribing guidelines from other countries (159/162, 98.1%). Most participants said they never chose an antibiotic based on what is cheaper (143/162, 88.3%) and based on trial and error (85/162, 52.5%). Further information on antibiotic supply and antibiotic choices can be found in Supplementary Tables 7, 8 respectively.

#### Antibiotic use profile in animals

More than half of the participants reported ever giving antibiotics to pigs, cattle, goat, buffalo, dogs and local chickens, and very few participants have ever used antibiotics in broilers and layers. Pigs (138/162, 85.2%), cattle (137/162, 84.6%) and buffalo (73/162, 45.1%) were selected in the top 3 species which participants administered antibiotics to in the last 12 months. Almost all participants (161/162, 99.4%) reported using antibiotics in pigs in the last 12 months. The classes of antibiotics that were used were tetracycline, penicillin, aminoglycoside and sulphonamides. The most used antibiotic by participants in pigs was Medoxy-LA (128/161, 79.5%). On the other hand, only 58.6% (95/162) of participants used antibiotics in chickens in the last 12 months. Of those participants that had used antibiotics in chickens in the last 12 months, the classes of antibiotics that were used were tetracycline, penicillin, aminoglycoside, sulphonamides and polypeptides. The most used antibiotic by participants in chickens was also Medoxy-LA (72/95, 75.8%). Further information on antibiotics use by species can be found in Supplementary Table 9.

#### Use of human antibiotics

Less than a fifth of participants (25/162, 15.4%) reported ever using human antibiotics in animals. Animal species that received human antibiotics from participants were local chickens, fighting cocks, pigs, dogs, cattle and goats. The human antibiotics given were mainly in tablet, capsule or ointment formulations. The common active ingredients were amoxicillin, ampicillin, tetracycline. The common brands mentioned were Super Tetra and Terramycin. The human antibiotics were mainly purchased from human pharmacies and kiosks. Several participants also reported using leftover human antibiotics that they had received for their own use from health centres/posts or hospitals. Further information on use of human antibiotics can be found in Supplementary Table 10.

#### Use of oral antibiotics

Less than 10% of participants reported ever giving antibiotics through water to animals, and such practices were mostly reported in municipalities bordering Indonesia. Chickens followed by pigs were the most mentioned species which were given antibiotics through water. The antibiotic brands that were mentioned contained amoxicillin, enrofloxacin, colistin, oxytetracycline, neomycin, ampicillin, sulphonamides and bacitracin as active ingredients. One participant mentioned giving an injectable formulation of oxytetracycline through water to chickens. Another participant used a poultry specific oral antibiotic in pigs and cattle. Only three participants reported giving antibiotics through feed to animals. While one could not recall the antibiotic given, the other two participants reported giving an injectable formulation of oxytetracycline through feed to pigs and dogs. Further information on use of oral antibiotics can be found in Supplementary Table 10.

#### Antibiotic prescribing practices

Before using antibiotics, more than three-quarter of participants (132/162, 81.5%) always checked the expiry date. When using antibiotics, around one-third of participants (52/162, 32.1%) reported ever giving a lower antibiotic dose than required on the label. Among these participants, the most stated reasons for giving a lower antibiotic dose were a sick or weak animal, inaccurate weight estimation and the lack of antibiotic supply. For participants who reported never giving a lower antibiotic dose than required on the label, the majority stated that it was important to adhere to label instructions. Slightly less than one-third of participants (48/162, 29.6%) reported ever giving an animal a shorter duration of antibiotic treatment than on the label. Among these participants, the common reasons for giving a shorter duration of antibiotic treatment were early animal recovery, lack of antibiotic supply, inability to revisit a distant farm to administer antibiotics and the farmer had released the animal. For participants who have never given a shorter duration of antibiotic treatment than recommended on the label, nearly all explained that they had to follow dosing instructions.

After giving antibiotics, most participants (123/162, 75.9%) always advised farmers to wait a few days before selling, slaughtering or eating products from their animals. When asked about what else was communicated to farmers when antibiotics were used, around half of the participants (76/162, 46.9%) also told farmers that that there was a possibility that animals might not improve and around a third of participants (55/162, 34.0%) advised farmers to implement good animal management. Less than 20% of participants advised farmers to isolate sick animals. None of the participants engaged with farmers on the impact of antimicrobial resistance and diagnostic testing. Most participants (146/162, 90.1%) recorded their antibiotic use on a paper-based form supplied by the MAF national office which was later submitted to the office for collation.

Less than a quarter of participants (38/162, 23.5%) reported that they have ever given farmers antibiotics to inject animals themselves, and only three participants reported that they have ever advised farmers to give antibiotics without first examining the animals themselves. Around half of participants (79/162, 48.8%) said they knew farmers who used antibiotics in animals without first speaking with a technician. When asked about the reasons for such a practise among farmers, participants cited poor access to veterinary services, the ability to purchase antibiotics from Indonesia or agriculture shops, and presence of knowledgeable farmers who administer antibiotics based on previous training. Further information on antibiotic prescribing practices can be found in Supplementary Table 11.

### Factors influencing knowledge and attitudes on antibiotic use and resistance

Exploratory analyses of the demographic variables identified that many were highly correlated and cross-tabulations showed some cells with very low frequencies. For example, position and education were highly correlated because all but two veterinary technicians had university education. Consequently, only crude logistic regression analyses were conducted but the causal diagrams informed discussion of the results and they and the rationale behind them are included for interest in Supplementary material.

#### Factors influencing knowledge

Veterinary technicians were more likely than livestock technicians (OR: 2.2; 95%Cl: 1.1−4.5), and those who attended university were more likely than those who did not attend university (OR: 2.8; 95%CI: 1.4−5.4) to have knowledge of how antibiotics work. A similar pattern was seen for knowledge of antibiotic resistance, where veterinary technicians were more likely than livestock technicians (OR: 3.4; 95%CI: 1.5−7.6), and those who attended university were more likely than those who did not attend university (OR: 2.9; 95%CI: 1.3−6.2) to know that antibiotic resistance reduces effectiveness of antibiotics. See Table 5 for regression analysis.

**Table 5:** Crude associations between demographic variables and knowledge of how antibiotics work and antibiotic resistance derived from a series of univariable logistic regression models.

	**Knowledge of how antibiotics work**		**Knowledge of antibiotic resistance**	
**Explanatory variable**	**OR (95% CI)**	***p***	**OR (95% CI)**	***p***
Position		***0.030***		***0.012***
Veterinary technician	2.2 (1.1–4.5)	**0.036**	3.4 (1.5–7.6)	**0.003**
Livestock technician	Ref		Ref	
Other	0.6 (0.2–1.5)	0.254	2.1 (0.6–7.0)	0.231
Age		*0.167*		*0.436*
<30	Ref		Ref	
30–39	2.6 (0.7–9.9)	0.160	0.4 (0.1–1.9)	0.275
40–49	1.2 (0.3–4.6)	0.817	0.3 (0.1–1.4)	0.127
≥50	1.4 (0.3–5.5)	0.664	0.3 (0.1–1.5)	0.160
Gender		*0.856*		*0.708*
Male	Ref		Ref	
Female	0.9 (0.3–2.5)	0.856	1.2 (0.4–3.7)	0.708
Experience		*0.353*		*0.701*
<10 yrs	Ref		Ref	
≥10 yrs	0.7 (0.4–1.4)	0.353	1.2 (0.5–2.6)	0.701
Highest education		***0.002***		***0.007***
Not university	Ref		Ref	
University	2.8 (1.4–5.4)	**0.002**	2.9 (1.3–6.2)	**0.007**
Antibiotic training		*0.114*		*0.247*
No	Ref		Ref	
Yes	0.6 (0.3–1.1)	0.114	0.6 (0.3–1.4)	0.247

OR: odds ratio, CI: confidence interval, overall likelihood ratio p-values in italics, individual Wald p-values in normal font, *p*-values < 0.05 are in bold.

#### Factors influencing attitudes

Exploratory analyses of some of the key knowledge variables suggested that the proposed causal diagram-based analyses were feasible. However, knowledge of antibiotic resistance was highly correlated with knowledge of antibiotic resistance transmission and antibiotic resistance as serious human and animal health issues. Furthermore, almost all participants knew that samples could be sent to the veterinary laboratory. Therefore, these variables were excluded from the analyses. The full causal diagram is shown in Supplementary material and the results from both total and direct effect analyses in Table 6. There was evidence of a positive total effect of knowledge of antibiotics (coefficient: 0.9; 95%CI: 0.4−1.4), antibiotic resistance (coefficient: 0.8; 95%CI: 0.3−1.4) and broad and narrow spectrum antibiotics (coefficient:1.1; 95%CI: 0.5−1.7) on attitude score. The direct effect of knowledge of antibiotics (coefficient: 0.5; 95%CI:−0.1−1.0) on attitude score was smaller than the total effect and imprecisely estimated. Residuals from each of the linear regression models fitted followed an approximately normal distribution.

**Table 6:** Estimated coefficients for the total and direct effects of key knowledge variables on attitude score derived from a series of linear regression models.

**Variable**	**Effect type**	**Coefficient (95% CI)**	***p***	**Covariates**
Knowledge of how antibiotics work	Total		**0.001**	None
No		Ref		
Yes		0.9 (0.4–1.4)		
Knowledge of how antibiotics work	Direct		0.096	Knowledge of antibiotic resistance, spectrum, critically important antimicrobials
No		Ref		
Yes		0.5 (−0.1–1.0)		
Knowledge of antibiotic resistance	Total/direct		**0.005**	Knowledge of how antibiotics work
No		Ref		
Yes		0.8 (0.3–1.4)		
Knowledge of critically important antimicrobials	Total/direct		0.074	Knowledge of how antibiotics work
No		Ref		
Yes		0.5 (0.0–1.0)		
Knowledge of spectrum	Total/direct		**<0.001**	Knowledge of how antibiotics work
No		Ref		
Yes		1.1 (0.5–1.7)		

*p*-values < 0.05 are in bold.

## Discussion

This is the first study describing the knowledge, attitudes and practices of antibiotic use and antibiotic resistance among government animal health workers in Timor-Leste, and one of few studies which explores this in an LMIC. This study found that GAHWs had poor knowledge on antibiotic and antibiotic resistance, but this was better among veterinary technicians and those with university education. Areas where prudent use of antibiotics could be improved were identified.

### Participant characteristic

Most veterinary technicians were under 40 years old, while most livestock technicians were 40 years or older. This reflects the more recent introduction of the veterinary technician position employing animal health graduates from the national university which produced graduates only after 2013, compared to the more historical livestock technician position. The increased female representation among veterinary technicians also reflects Timor-Leste's ongoing efforts to achieve gender equality (41).

### Poor knowledge on antibiotics and antibiotic resistance

Knowledge of antibiotics among animal health workers was relatively poor compared to other LMICs. The proportion of participants who correctly identified that antibiotics did not kill or inhibit viruses in this study (59.9%) was lower than Bhutan (76.5%) (9) and Nigeria (93.4%) (35). Furthermore, a large proportion of participants in this study incorrectly identified that antibiotics had direct anti-inflammatory (87.0%) and anti-pyretic effects (86.4%). This was even higher than the general adult population in other LMICs, where 9.7% of participants in Jordan and 63.5% of participants in Indonesia incorrectly identified that antibiotics had anti-pyretic properties (42, 43). While it was positive that almost all participants were able to identify Medoxy-LA as an antibiotic, almost half of participants thought that ivermectin was an antibiotic, indicating that GAHWs may find it difficult to differentiate anti-parasitics from antibiotics.

Knowledge of antibiotic resistance was poor among GAHWs in Timor-Leste as only 29.0% of participants had heard of antibiotic resistance and were able to accurately identify that it made antibiotics less effective. This was much lower than GAHWs in Bhutan where almost all participants (95.4%) indicated that resistant bacteria are difficult to treat (9), although the participants in the Bhutan study included a small proportion of veterinarians. It was however positive that the knowledge of antibiotic resistance among GAHWs was higher than smallholder pig farmers in Timor-Leste, where none of the farmers could explain the concept of antibiotic resistance (34). There was low awareness of the efforts to combat antibiotic resistance, with less than a third having knowledge of the National Action Plan for Antimicrobial Resistance compared to 59.8% for the equivalent in Nigeria (35).

To address the poor knowledge, future MAF training which GAHW identified as their most common source of knowledge could focus on addressing the identified gaps such as emphasising that antibiotics do not have direct anti-inflammatory or anti-pyretic properties, identification of common veterinary medicines and the concept of antibiotic resistance. Trainings should focus on municipalities such as Oecusse and Ainaro where there is poorer knowledge.

Knowledge of antibiotics and knowledge of antibiotic resistance were all crudely associated with being a veterinary technician rather than a livestock technician and having university education. These findings are consistent with a study conducted in Bhutan, where GAHWs with at least university education had better overall scores for knowledge on antibiotics and antibiotic resistance (9). Studies among animal health workers in other LMICs have also found level of education to be a factor influencing knowledge of antibiotic and antibiotic resistance (27, 35, 44). We had hoped to conduct a causal-diagram informed regression analysis that might have enabled us to better estimate the true total and direct effects of each of these demographic variables by reducing confounding and teasing apart the causal pathways. However, this was not possible because most demographic variables were highly correlated. More specifically, position and education were highly correlated because all but two veterinary technicians had university education.

### Attitudes on antibiotic use

This study found that veterinary technicians (who are younger in age) had a higher median attitude score compared to non-veterinary technicians. This is consistent with a study in India which showed that those under 30 years old had higher attitude scores (44).

Although nearly all participants believed that laboratory results can help with decisions on antibiotics use, this did not translate into practise as sample submission to the laboratory by participants was low, most have never received a laboratory result and almost all participants have never chosen an antibiotic based on laboratory results. Though the reasons for the low sample submission was not investigated in this study, low sample submission to laboratories by animal health workers in other LMICs is often due to limited access to laboratory services (8, 36, 44). Therefore, improving access to laboratory services for GAHWs may help GAHWs to make better antibiotic use decisions.

It was positive that the majority of participants believe that the use of vaccines, implementation of farm biosecurity, and practise of good animal husbandry can reduce antibiotic use in Timor-Leste. However, farm observation by participants in this study showed that vaccine coverage is suboptimal and farm biosecurity practices are typically low in smallholder farms, which is in agreement with previous studies (45–47). Although there are ongoing projects to improve vaccination, husbandry and biosecurity in Timor-Leste, many of these target selected areas or farmer groups (21, 26, 48, 49), and nationwide expansion of these initiatives are required.

The use of antibiotics for growth promotion, especially those that are medically important is discouraged and banned in many countries (50–52). Although a fifth of participants believed in giving healthy animals antibiotics to promote growth, such attitudes did not reflect actual practise, where only six participants have ever used antibiotics for growth promotion. This may reflect the nature of technician work, where they are usually called out to visit farms with disease problems instead of improving productivity. Most antibiotics are also provided in injectable form instead of oral which reduces their use for growth promotion. Future training programmes for GAHWs should include a component to address the believe that antibiotics should be used to promote growth.

It is worrying that around three-quarters of participants believed that giving a broad spectrum antibiotic was always a better choice than a narrow spectrum antibiotic, which was very similar to a finding in Bhutan where 72% of respondents indicated that broad spectrum antibiotics should be used for any bacterial infection (9). This is likely due to GAHWs selecting an antibiotic based on the objective of maximising the likelihood of the antibiotic being effective against the infection in the absence of diagnostic support, rather than considering the impact of this approach on antibiotic resistance. The development of an antibiotic guidelines based on national and international sensitivity patterns to different pathogens could assist GAHW to select narrow spectrum antibiotics for first line treatment.

The positive total effects of knowledge of how antibiotics work, antibiotic resistance and spectrum of antibiotics on attitude score was encouraging as it suggests that efforts to improve knowledge levels of the GAHW population will translate to an improvement in their attitudes towards antibiotic use and hopefully also their practices. However, the direct effect of knowledge of antibiotics was lower and less precisely estimated, suggesting that a deeper level of knowledge including knowledge of antibiotic resistance and spectrum of antibiotics is important to positively influence attitude score.

### Characterising antibiotic use practices

It is of concern that most participants always gave antibiotics to sick animals, where the antibiotics were used empirically to treat any case of diarrhoea, fever, cough or swelling even though the aetiology might not be bacterial. Such a practise was also reported among GAHWs elsewhere (9) and could be due to the poor knowledge on antibiotics and antibiotic resistance reported earlier. Another contributor to such a practise could be pressure from owners to administer antibiotics as some participants reported facing resistance from farmers when deciding not to use antibiotics. The pressure to administer antibiotics exerted by animal owners has also been found in other studies (12, 27, 44, 53). Another reason for the empiric use of antibiotics in all sick animals could be due to a lack of diagnostic support for GAHWs. This again highlights a need to improve diagnostic support for GAHWs in Timor-Leste, especially in a manner which allows them to receive results in a timely manner to inform treatment choices. Diagnostic support also allows GAHWs to identify and address the underlying cause of disease that results in secondary bacterial infections which will avoid repeated use of antibiotics that increases the risk of resistance development (54). Separately, it was concerning that participants reported giving antibiotics inappropriately for non-bacterial infections such as scabies and worms, although it could be argued that antibiotics were given to prevent secondary bacterial infections in some cases. Development of Timor-Leste specific treatment guidelines accompanied by training sessions could be useful in addressing the inappropriate use of antibiotics in sick animals, as the development of such guidelines has proved to be effective in influencing antibiotic choice (35) and promoting prudent use in other countries (55–58).

There were a myriad of factors influencing the choice of antibiotic. Factors such as past experience and availability of drugs which the majority of participants always used to select antibiotics was similar to findings in other LMICs (9, 36, 44, 59). The majority of participants never chose an antibiotic based on cost and this was different to findings in other studies (12, 35, 44, 60). The absence of cost considerations is likely due to veterinary medicines usually being provided free-of-charge by GAHWs to farmers in Timor-Leste (34). Almost all participants never considered choosing an antibiotic based on a laboratory test or antibiotic prescribing guidelines, which reflects the current state where there is limited laboratory access and no prescribing guidelines specific to the country. The majority of participants always selected antibiotics based on their duration of action, which reflects the preference for GAHWs to administer long-acting antibiotics as farms in remote locations are hard to revisit and free-grazing animals are hard to recapture for follow-up treatment.

The lack of supply of antibiotics and other veterinary medicines found in this study was consistent with another report (20), and similar challenges has been reported in other LMICs (9, 12, 61). The lack of supply could be the reason why some technicians turned to alternative sources such as agriculture shops for antibiotics, while others have used human antibiotics. This was also a common reason why technicians reported administering a lower antibiotic dose and shorter treatment duration, which can result in ineffective treatment and increase the risk of resistance developing (62, 63). Efforts are currently being made by Menzies under the Fleming Fund Country Grant to collect more granular information on disease and antibiotic use patterns in the country (24), which can be used to improve veterinary medicines procurement decisions to meet animal health needs in the country.

There was a limited range of antibiotics available to GAHWs, and similar findings have been reported in other LMICs (12, 59). The classes of antibiotics commonly used by technicians in animals in this study such as tetracycline are consistent with previous studies in Timor-Leste (25, 34). Prior to this study, it was thought that the species which most frequently received antibiotics from GAHWs were pigs and local chickens because they are the most owned species by households (19). Therefore, the survey dedicated sections on understanding antibiotic use in both these species. This study however revealed that cattle and buffalo were rated by many GAHWs as among the top three species to which they administered antibiotics and future studies could evaluate antibiotics use practices in these animals. It was also revealed in this study that broilers and layer chickens rarely received antibiotics from GAHWs, suggesting that broilers and layers are usually reared in larger scale farms that likely rely on non-government animal health workers to administer antibiotics. Further investigation on antibiotic usage in broiler and layer farms would be useful in future studies.

The mass administration of antibiotics orally *via* water or feed over a prolonged duration increases the selection pressure for resistance especially for gastrointestinal bacteria (55, 64). Improper dosing is also common due to non-homogeneous mixing, insolubility of the antibiotic or reduced water or feed consumption in sick animals (52). It was encouraging that the use of oral antibiotics among participants was uncommon in this study, and most participants did not recognise the oral antibiotics that were commonly imported into the country (25). Participants who have ever used oral antibiotics were mainly from municipalities closer to Indonesia, suggesting that this practise may have been introduced from Indonesia. This study also found that some GAHWs were administering antibiotics through an incorrect route by adding injectable formulations of oxytetracycline in feed and water which can affects its absorption and bioavailability (65, 66). The use of human oral antibiotics in animals was reported by some participants in this study, which has also been observed in other LMICs (51, 67). The misuse of antibiotics through incorrect route of administration and species of use could be addressed through future training sessions.

The current situation of remote farms and free-grazing animals coupled with limited manpower challenges the capacity of the veterinary service in Timor-Leste to provide quality treatment services, which has also been reported in other LMICs (9, 12, 27). This could explain why several participants reported providing farmers with antibiotics to inject animals themselves in this study, which increases the likelihood of misuse especially if no instructions were given. There are ongoing efforts to strengthen the field veterinary service, including plans to increase the number of government-employed veterinarians substantially over the next 2 years (23).

### Strengths and limitations

This study has several strengths. Our findings provided an understanding of the KAP of the largest group of veterinary antibiotic users in the country, which uses more than 50% of the veterinary antibiotics available in the country. Furthermore, the census-based approach means that there can be very little random error in our estimates compared to a study that selected a sample from the target population. Strong coordination and collaboration between Menzies and MAF, together with a respectful approach likely contributed to the high participation rate in this study. Piloting of the questionnaire improved clarity and reduced the likelihood of questions being misunderstood especially as it was initially written in English but administered in Tetun. However, there is still a small risk of misclassification bias especially for certain concepts such as withholding periods which did not have direct translations and had to be expressed in a convoluted manner. The use of picture aids for antibiotic products was also an important strategy to stimulate participants' memories and reduce recall bias.

One limitation of our study was the inability to interview the entire target population as we had planned. However, selection bias was limited as participation was high at 89.9% and the main reason for non-participation was work commitments. Another limitation of our study was that it did not capture the KAP of other users of veterinary antibiotics, such as private animal health workers on commercial farm properties which account for around a third of veterinary antibiotic use. The KAP among these users could be investigated in future studies. We acknowledge the likelihood of some response bias as some participants may have provided an answer that they thought was desirable. We sought to minimise this by reassuring participants that their responses were anonymous and interviews were conducted in a judgement-free manner.

## Conclusion

This study showed that knowledge of antibiotics and antimicrobial resistance was poor among GAHWs, with many GAHWs incorrectly stating that antibiotics had anti-viral, anti-inflammatory and anti-pyretic properties. It also revealed that veterinary technicians and those with university education were more likely to have knowledge of how antibiotics work and antimicrobial resistance.

Attitude scores on antibiotics use were found to be influenced by knowledge, and it was concerning that almost all GAHWs believed that broad spectrum antibiotics were always a better choice narrow spectrum antibiotics.

Pigs, cattle and buffalo were rated by most GAHWs as among the top three species to which they administered antibiotics, and oxytetracycline was the most used antibiotic in pigs and chickens. Empiric use of antibiotics in sick animals was common, likely due to the lack of diagnostic support. Many GAHWs reported a lack of antibiotic supply, with some responding by giving a lower dose or shorter duration treatment in animals. The use of oral antibiotics was mainly in municipalities close to Indonesia, with some GAHWs administering injectable formulations orally or using human oral antibiotics.

The development of treatment guidelines specific to Timor-Leste, strengthening of veterinary diagnostic support, improving antibiotic procurement to be responsive to disease needs, and training programmes to address knowledge gaps and poor practices is recommended.

## Data availability statement

The datasets generated in this study are available on request from the corresponding author.

## Ethics statement

Ethics approval for this research was obtained from the Human Research Ethics Committee of the Northern Territory (NT) Department of Health and Menzies School of Health Research (2020-3841) and Institute Nacional de Saude in Timor-Leste (MS-INS/DE/IX/2020/1411). An information sheet in the Tetun language was provided and verbally explained to all participants prior to the interview. Informed consent was given by the participant via a written signature. Participants were given the right to decline participation or withdraw from the interview at any stage. Actions were taken to ensure confidentiality and anonymity of interview responses.

## Author contributions

ST, AP, AA, PV, CD, JJ, SD, JY, JF, and TB conceived and designed the study. AP, AA, PV, and CD conducted the field work. ST, TB, AP, SD, AA, CD, and PV analysed the data. ST, AA, TB, and SD prepared the draft manuscript. JY, JF JJ, SD, HS, AP, AA, CD, and PV reviewed and edited the manuscript. All authors have read and approved the final manuscript.

## Funding

This study was supported by the Fleming Fund Country Grant for Timor-Leste (FF/87/493). The Fleming Fund is a UK aid investment programme to tackle antimicrobial resistance in LMICs around the world and is managed by the UK Department of Health and Social Care.

## Conflict of interest

Author TB is a working director of Epivet Pty. Ltd. The remaining authors declare that the research was conducted in the absence of any commercial or financial relationships that could be construed as a potential conflict of interest.

## Publisher's note

All claims expressed in this article are solely those of the authors and do not necessarily represent those of their affiliated organizations, or those of the publisher, the editors and the reviewers. Any product that may be evaluated in this article, or claim that may be made by its manufacturer, is not guaranteed or endorsed by the publisher.
