# Supplementary figures and images for: Knowledge, attitudes and practices of government animal health workers on antibiotic use and antibiotic resistance in Timor-Leste
Source: Front Vet Sci. 2022 Nov 24;9:1063530. doi: 10.3389/fvets.2022.1063530 (PMC9731573; doi:10.3389/fvets.2022.1063530)

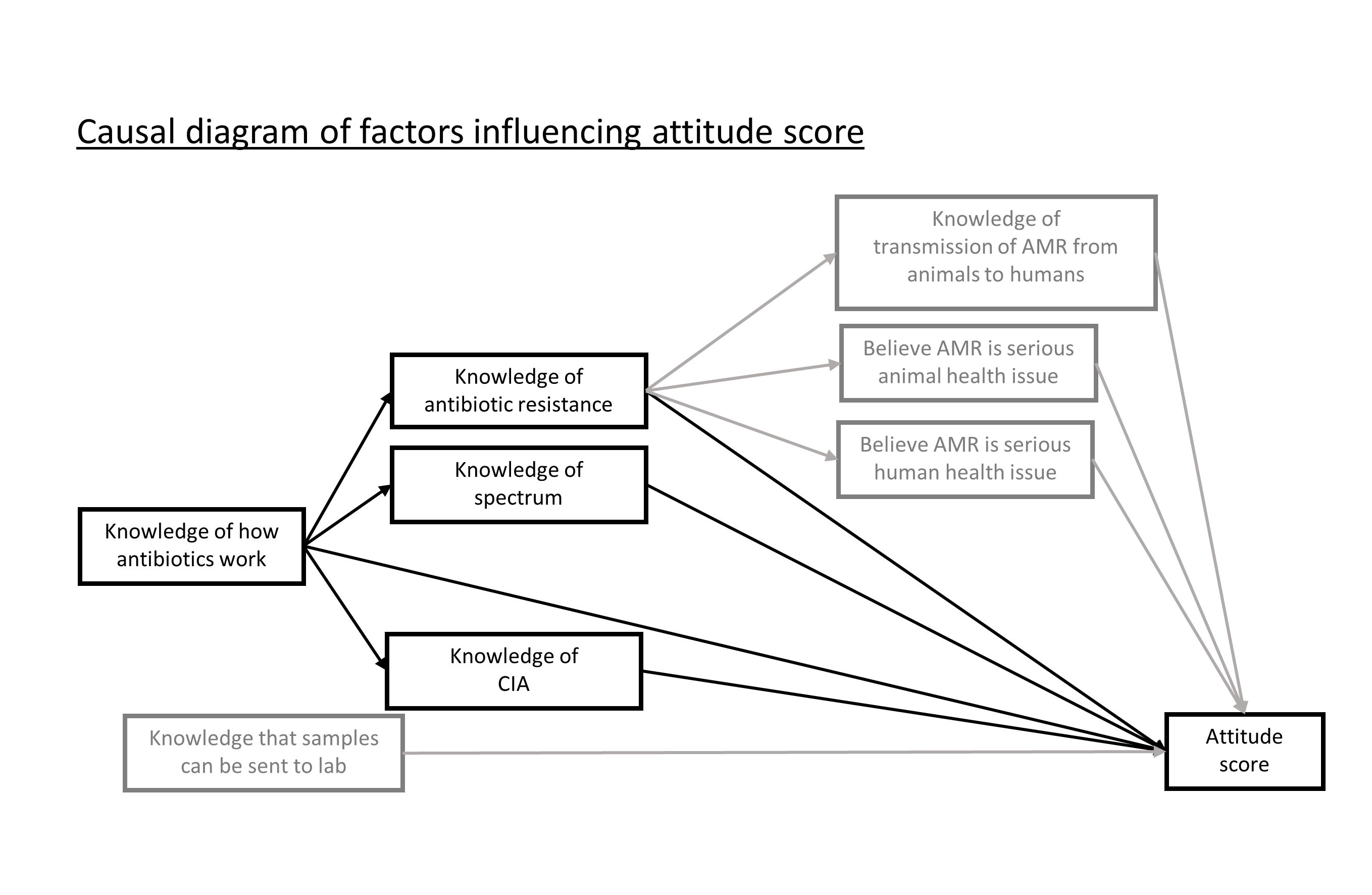

Supplement: Supplementary file 1 [file Image_1.TIF]
