# Supplementary material for: Knowledge, attitudes and practices of government animal health workers on antibiotic use and antibiotic resistance in Timor-Leste
Source: Front Vet Sci. 2022 Nov 24;9:1063530. doi: 10.3389/fvets.2022.1063530 (PMC9731573; doi:10.3389/fvets.2022.1063530)
Supplement: Supplementary file 2 [file Data_Sheet_1.PDF]

Knowledge, attitudes and practices on antibiotic and antibiotic resistance in Timor-Leste

GOVERNMENT ANIMAL HEALTH WORKER INTERVIEW

Section 1 – Demographics of participant and area

Interviewer name ____

Date of interview_________________ **Municipality** _____________________

Respondent name ____

What is your mobile number ____

1. Gender**:** ◻ Male ◻ Female
2. Age____ years

3. What is your highest education level? (Select only one)

   - ◻ University (animal science)
   - ◻ University (animal health)
   - ◻ University (not animal science or animal health)
   - ◻ Senior high school (agriculture school)
   - ◻ Senior high school (non-agriculture school)
   - ◻ Junior high school
   - ◻ Others ___________________

4. Where did you receive your highest education? (Select only one)

   - ◻ Timor-Leste
   - ◻ Indonesia
   - ◻ Others: _________

5. What is your position (Select only one)

   - ◻ Veterinary technician
   - ◻ Livestock technician
   - ◻ Extension worker
   - ◻ Others: ____________

6. How many years have you worked as a vet/livestock technician (round up to nearest year) _______ years

6b. How many years have you worked as a vet/livestock technician in the field. ["Field" means being in a role that involves being called out to provide animal health services such as treatment or vaccination on a regular basis] _______ years

7. Which posto administrativo do you live in? ___________

8. Where do you *usually* work from? (Select one only)
    - □ Home
    - □ Municipal office
    - □ Posto administrativo office
    - □ Animal Health Centre
    - □ National Directorate of Veterinary
    - □ Animal Production Centre
    - □ Extension Centre

9. Are you assigned to one or more posto administrativo?
    - □ Yes
    - □ No

10. If yes, which posto administravo are you assigned to? [Tick all relevant]

11. How many "luhans or farms' have you visited for work on average per month for the last 12 months before COVID-19 sanitary fencing (i.e. February 2020 to February 2021)? _____ luhans or farms
    [Definitions: Farm refers to a property with commercial animal farming; Luhan refers to a property with backyard animal farming]

12. What were the purposes of the "luhan or farm" visits that you made in the last 12 months before COVID-19 sanitary fencing (from February 2020 to February 2021)? [Tick all relevant]

    - □ Vaccination
    - □ Treatment of sick animals
    - □ Give advice to farmer on livestock disease
    - □ Give advice to farmer on livestock management (e.g. feeding and housing)
    - □ Others: ______

13. What percentage of chickens do you think have up to date Newcastle Disease vaccination in the posto administrative(s) that you cover for work? (Select only one)
    - □ 0% – 33%
    - □ 34% – 66%
    - □ 67% - 100%
    - □ Don't know

14. What percentage of pigs do you think have up to date CSF vaccination in the posto administrative(s) that you cover for work? (Select only one)
    - □ 0% – 33%
    - □ 34% – 66%
    - □ 67% - 100%
    - □ Don't know

15. Have you observed fencing around the property during visits to “farms or luhans” with chickens and pigs?
    - □ Never
    - □ Occasionally
    - □ Frequently

16. Have you observed locked gates on properties during visits to “farms or luhans” with chickens or pigs?
    - □ Never
    - □ Occasionally
    - □ Frequently

17. Have you observed chickens in cages during visits to “farms or luhans”?
    - □ Never
    - □ Occasionally
    - □ Frequently

18. Have you observed other animals in pens during visits to “farms or luhans”?
    - □ Never
    - □ Occasionally
    - □ Frequently

19. Have you observed farmers wearing dedicated boots when tending to animals during visits to “farms or luhans”?
    - □ Never
    - □ Occasionally
    - □ Frequently

20. Have you observed disinfection of footwear during visits to “farms or luhans”?
    - □ Never
    - □ Occasionally
    - □ Frequently

21. Have you seen visitor sign-in books during visits to “farms or luhans”? *[Definition: A visitor sign-in book is used to record the names of visitors that visit a property]*
    - □ Never
    - □ Occasionally
    - □ Frequently

22. Do you know that samples from *sick* animals can be sent to the laboratory for diagnosis?
    - □ Yes
    - □ No

23. If yes, have you *ever* sent samples from *sick* animals to the laboratory for diagnosis?
    - □ Yes
    - □ No

24. If yes, how many samples from *sick* animals have you collected and sent to the laboratory in the last 12 months before COVID-19 sanitary fencing (i.e. February 2020 to February 2021)? __________ samples from sick animals

25. If yes, how often do you receive a lab test result from the laboratory? (Select only one)
    - ◻ Always
    - ◻ Sometimes
    - ◻ Never

Section 2 – Knowledge about antibiotics and antibiotic resistance

Before starting on this section state "Just to let you know beforehand that this section will have more technical questions that you might find difficult. Don't feel embarrassed if you do not know the answers as it is not a test. The responses will help Menzies to improve training on antibiotic use for veterinary and livestock technicians. There are no personal consequences to your responses and they will be kept confidential and unidentifiable."

26. Do you know what antibiotic medicine for animal are? [Select only one]

- ◻ Yes
- ◻ No

If "No" - state that "Antibiotics is a medicine used to treat sick animals with bacteria infection" Then, proceed to "Have you heard what is antibiotic resistance (Qn 37)".

27. If yes, where did you learn about antibiotic medicine for animals use [Tick all relevant]

- ◻ University
- ◻ Senior high school
- ◻ Colleagues
- ◻ MAF training course
- ◻ Non-MAF training course
- ◻ Others: __________

If yes, answer question 28 to 32 with True / False / Don't know

28. Antibiotics kills or inhibits *virus*

- ◻ True
- ◻ False
- ◻ Don't know

29. Antibiotics kills or inhibits *bacteria*

- ◻ True
- ◻ False
- ◻ Don't know

30. Antibiotics kills or inhibits *parasites*

- ◻ True
- ◻ False
- ◻ Don't know

31. Antibiotics *directly* reduces inflammation in animals

- ◻ True
- ◻ False
- ◻ Don't know

32. Antibiotics *directly* reduces fever in animals

- ◻ True
- ◻ False
- ◻ Don't know

<u>If any of the questions 28 - 32 is wrong</u> - state that "Antibiotics is a medicine used to treat sick animals with bacteria infection". Then, proceed to next question.

33. If yes, which of the following are antibiotics? [Show pictures]

- ◻ Vitamin B Complex [Yes / No / Don't know]
- ◻ Medoxy-LA [Yes / No / Don't know]
- ◻ Ivermectin [Yes / No / Don't know]
- ◻ Albendissu (Albendazole) [Yes / No / Don't know]
- ◻ Pen-strep 400 [Yes / No / Don't know]
- ◻ Biosolamine [Yes / No / Don't know]
- ◻ Sulfa strong (sulfabac) [Yes / No / Don't know]
- ◻ Novaldon [Yes / No / Don't know]
- ◻ Gusanex [Yes / No / Don't know]
- ◻ Colibact [Yes / No / Don't know]
- ◻ Oxytetracylcine HCL [Yes / No / Don't know]

34. Which of these vet medicines have been lacking in supply for use in animals as part of your work in the last 12 months [Show pictures – Please note that pictures are only an example]

- ◻ Medoxy-LA [Yes / No / Don't know]
- ◻ Ivermectin [Yes / No / Don't know]
- ◻ Albendissu (Albendazole) [Yes / No / Don't know]
- ◻ Pen-strep 400 [Yes / No / Don't know]
- ◻ Sulfa strong (sulfabac) [Yes / No / Don't know]
- ◻ Colibact [Yes / No / Don't know]
- ◻ Gusanex [Yes / No / Don't know]
- ◻ Intramox [Yes / No / Don't know]
- ◻ Trypamidium [Yes / No / Don't know]
- ◻ Oxytetracylcine HCL [Yes / No / Don't know]

35. Have you *ever* obtained antibiotics from sources other than MAF for use in your work?

- ◻ Yes
- ◻ No

36. If yes, where did you get antibiotics from other than from MAF? [Tick all relevant]

- ◻ Agriculture shop
- ◻ Market
- ◻ Pharmacy
- ◻ Others: Please specify ____

37. Have you heard of antibiotic resistance? (Select only one)

- ◻ Yes
- ◻ No

38. If yes, do you know what is antibiotic resistance? (Select only one)

- ◻ Yes
- ◻ No

<u>**If "No"**</u> – skip to question 41.

39. If yes, where did you learn about antibiotic resistance [Tick all relevant]

- ◻ University
- ◻ Senior high school
- ◻ Colleagues
- ◻ MAF training course
- ◻ Non-MAF training course
- ◻ Others: __________

40. If yes, how does antibiotic resistance change the effectiveness of antibiotics? [select one] [read options]

- ◻ Antibiotic is less effective
- ◻ Antibiotic is more effective
- ◻ No change to effectiveness
- ◻ Don't Know

<u>**If question 40 is wrong**</u> - state that "Antibiotic resistance makes antibiotics less effective". Then, proceed to next question.

41. Have you heard of the need to wait a few days after giving antibiotics before slaughtering or selling animals?

- ◻ Yes
- ◻ No

42. Have you heard of the risk of antibiotic residues in animal or animal products after antibiotics are used?
    - ◻ Yes
    - ◻ No

43. Have you heard of broad and narrow spectrum antibiotics?
    - ◻ Yes
    - ◻ No

44. If yes, is Medoxy a broad or narrow spectrum antibiotic?
    - ◻ Broad spectrum
    - ◻ Narrow spectrum
    - ◻ Don’t know

45. If yes, is Penstrep a broad or narrow spectrum antibiotic?
    - ◻ Broad spectrum
    - ◻ Narrow spectrum
    - ◻ Don’t know

46. If yes, is sulfa-strong / sulfabac a broad or narrow spectrum antibiotic?
    - ◻ Broad spectrum
    - ◻ Narrow spectrum
    - ◻ Don’t know

47. Can antibiotic resistance in animals lead to antibiotic resistance in humans?
    - ◻ Yes
    - ◻ No
    - ◻ Don’t know

48. Is antibiotic resistance a serious human health issue
    - ◻ Yes

- ◻ No
- ◻ Don’t know

49. Is antibiotic resistance a serious animal health issue
    - ◻ Yes
    - ◻ No
    - ◻ Don’t know

50. Have you heard of the World Antimicrobial Awareness Week?
    - ◻ Yes
    - ◻ No

51. Have you heard of the National Action Plan for Antimicrobial Resistance for Timor-Leste
    - ◻ Yes
    - ◻ No

52. Have you heard of critically important antimicrobial for human medicine?
    - ◻ Yes
    - ◻ No

Section 3 – Practice of antibiotic use in animals

It must be clearly established what is and is not an antibiotic before proceeding with this section.

State that "Antibiotics is a medicine used to treat or prevent bacteria infection."

53. Have you *ever* used antibiotics in animals for work? (Select only one option)

    - ◻ Yes
    - ◻ No
    - ◻ Don't know

54. **If "No"**. Why do you not use antibiotics to animals? [Tick all relevant]

    - ◻ Insufficient supply from MAF
    - ◻ Expensive
    - ◻ Not available nearby
    - ◻ Not effective
    - ◻ Others: ____

55. If **"Yes",** how many animals have you given antibiotics on average per month, in the last 12 months before COVID-19 sanitary fencing (i.e. February 2020 to February 2021)?

56. Have you *ever* used antibiotics in sick animals to help them recover?

    - ◻ Yes
    - ◻ No
    - ◻ Don't know

57. Have you *ever* used antibiotics in *healthy* animals that have been in contact with sick animals?

    - ◻ Yes
    - ◻ No
    - ◻ Don't know

58. Have you *ever* used antibiotics to help *healthy* animals grow faster?

    - ◻ Yes
    - ◻ No
    - ◻ Don't know

59. Have you *ever* given antibiotics through water to animals?

- ◻ Yes
- ◻ No
- ◻ Don’t know

60. If yes, which animal did you give antibiotics through water? [Tick all relevant]

- ◻ Local chicken
- ◻ Broiler
- ◻ Layer
- ◻ Fighting cock
- ◻ Pig
- ◻ Other: _________

61. If yes, what antibiotic(s) was given to animals through water? ____________________

62. Have you *ever* observed antibiotics given to animals through water in Timor-Leste?

- ◻ Yes
- ◻ No
- ◻ Don’t know

63. Have you *ever* given antibiotics through feed to animals?

- ◻ Yes
- ◻ No
- ◻ Don’t know

64. If yes, which animal did you give antibiotics through feed? [Tick all relevant]

- ◻ Local chicken
- ◻ Broiler
- ◻ Layer
- ◻ Fighting cock
- ◻ Pig
- ◻ Other: _________

65. If yes, what antibiotic(s) was given to animals through <u>feed</u>? ____________________

66. Have you *ever* observed antibiotics given to animals through feed in Timor-Leste?

- ◻ Yes
- ◻ No
- ◻ Don't know

67. Have you *ever* given human antibiotics to animals?

- ◻ Yes
- ◻ No
- ◻ Don't know

68. If yes, which animal species did you give human antibiotics to? [Tick all relevant]

- ◻ Local chicken
- ◻ Broiler
- ◻ Layer
- ◻ Fighting cock
- ◻ Pig
- ◻ Cattle
- ◻ Buffalo
- ◻ Goat
- ◻ Dog
- ◻ Cat
- ◻ Others:

69. If yes, which human antibiotic(s) did you give to animals? ____________________

70. If yes, where was the antibiotic obtained from? ___________________

71. Have you *ever* observed human antibiotics given to animals in Timor-Leste? [Select one]

- ◻ Yes
- ◻ No
- ◻ Don't know

72. Do you *always* give antibiotics to a sick animal? [Select one]

- ◻ Yes
- ◻ No

73. If no, what were the reasons that you decided <u>not</u> to use antibiotics in a sick animal [Read out] [Tick all relevant]

- ▢ No antibiotics available
- ▢ No appropriate antibiotic available
- ▢ Conserving antibiotics because there is a low supply of antibiotics left
- ▢ Dying (moribund) animal
- ▢ Low value animal
- ▢ Animal unlikely to have bacteria infection
- ▢ Do not want to be blamed by farmer for causing death of animal
- ▢ Residues in meat if animal dies before withholding period ends
- ▢ Others: ____________________

74. If no, have you faced resistance from the farmer for deciding not to use antibiotics in a sick animal?

- ▢ Yes
- ▢ No

Which of the following do you consider when choosing which antibiotics to use in a *sick* animal? (Qn 78 to 90)

75. I decide on which antibiotic to use in a *sick* animal based on my past experience on what has worked for the suspected disease (Always / sometimes / never)
76. I decide on which antibiotic to use in a *sick* animal based on my colleague's experience on what has worked for the suspected disease (Always / sometimes / never)
77. I decide on which antibiotic to use in a *sick* animal based on what I learnt at university or high school (Always / sometimes / never)
78. I decide on which antibiotic to use in a *sick* animal based on what I learnt at a post-graduation training course (Always / sometimes / never)
79. I decide on which antibiotic to use based on prescribing guidelines from other countries (Always / sometimes / never)
80. I decide on which antibiotic to use in a *sick* animal based on what is available (Always / sometimes / never)
81. I decide on which antibiotic to use in a *sick* animal based on which is cheaper (Always / sometimes / never)
82. I decide on which antibiotic to use in a *sick* animal based on whether the drug label states the antibiotic is effective for the suspected disease (Always / sometimes / never)
83. I decide on which antibiotic to use in a *sick* animal based on duration of action of the antimicrobial (Always / sometimes / never)

84. I decide on which antibiotic to use in a *sick* animal based on how long the meat/milk/eggs from the animal should not be eaten after giving the antibiotic (Always / sometimes / never)

85. I decide on which antibiotic to use in a *sick* animal based on laboratory test result (Always / sometimes / never)

86. I decide on which antibiotic to use in a *sick* animal based on trial and error (Always / sometimes / never)

87. Are there any other criteria that you consider when choosing which antibiotic to use in a *sick* animal? ____________________

88. What clinical signs in sick animals will prompt you to use *antibiotic* [Tick all relevant] [If answered "yes" to question 56 on "Have you *ever* used antibiotics in sick animals to help them recover "]

- ◻ Diarrhoea
- ◻ Fever
- ◻ Respiratory signs
- ◻ Skin infection
- ◻ Others: ____

89. What other options do you use for treating a sick animal

    a. Anti-inflammatory
    b. Vitamin B complex
    c. Herbal medicine
    d. Anti-parasitics or anti-protozoal
    e. Others: _______________

90. Which animal species have you *ever* used antibiotics on? This includes all routes of administration. [Tick all relevant]

- ◻ Local chicken
- ◻ Broiler
- ◻ Layer
- ◻ Fighting cock
- ◻ Pig
- ◻ Cattle
- ◻ Buffalo
- ◻ Goat
- ◻ Dog

- ◻ Cat
- ◻ Others: ____

91. What are the top 3 species do you use antibiotics on in the *last 12 months*? [Tick only 3] [This refers to the top 3 species animal species that antibiotics is most frequently administered to. Not the number of visits.]
    - ◻ Local chicken
    - ◻ Broiler
    - ◻ Layer
    - ◻ Fighting cock
    - ◻ Pig
    - ◻ Cattle
    - ◻ Buffalo
    - ◻ Goat
    - ◻ Dog
    - ◻ Cat
    - ◻ Others: ____

92. Which antibiotics have you used in chickens (manu local, broiler, layer) in the last 12 months? This includes all routes of administration. [Tick all relevant]
    - ◻ Penstrep
    - ◻ Medoxy-LA
    - ◻ Sulfa-strong (sulfabac)
    - ◻ Oxytetracycline HCL (Salip Mata)
    - ◻ Don’t know
    - ◻ Don’t use antibiotics
    - ◻ others (especially if used in feed and water): ____ ____

93. Which antibiotic (from question above) have *you* used <u>most frequently</u> in chickens in the last 12 months (manu local, broiler, layer)? [Select only one]
    - ◻ Penstrep
    - ◻ Medoxy-LA
    - ◻ Sulfa-strong (sulfabac)

- ◻ Oxytetracycline HCL (Salip Mata)
- ◻ others (especially if used in feed and water): ______________________________

94. Which antibiotics have you used in pigs in the last 12 months? [Tick all relevant]
    - ◻ Penstrep
    - ◻ Medoxy-LA
    - ◻ Sulfa-strong (sulfabac)
    - ◻ Oxytetracycline HCL (Salip Mata)
    - ◻ Don’t know
    - ◻ Don’t use antibiotics
    - ◻ others (especially if used in feed and water): ______________________________

95. Which antibiotic (from question above) have *you* used <u>most frequently</u> in pigs in the last 12 months? [Select only one]
    - ◻ Penstrep
    - ◻ Medoxy-LA
    - ◻ Sulfa-strong (sulfabac)
    - ◻ Oxytetracycline HCL (Salip Mata)
    - ◻ others (especially if used in feed and water): ______________________________

96. Have you *ever* used the following antibiotics in animals? [Show pictures]
    - ◻ Colistin [Yes / No / Don’t know]
    - ◻ Tylosin [Yes / No / Don’t know]
    - ◻ Amoxitin [Yes / No / Don’t know]
    - ◻ Koleridine [Yes / No / Don’t know]
    - ◻ Ampicol [Yes / No / Don’t know]
    - ◻ Entrocolin [Yes / No / Don’t know]
    - ◻ Cola Floxa [Yes / No / Don’t know]
    - ◻ Benmoxyl 50s [Yes / No / Don’t know]
    - ◻ Interflox [Yes / No / Don’t know]

97. Do you read the drug label instructions when using antibiotics? [Tick only one]

- ◻ Always
- ◻ Sometimes
- ◻ Never

98. Have you *ever* given an animal a lower antibiotic dose than recommended on the label (or product leaflet)?

- ◻ Yes
- ◻ No
- ◻ Don’t know

99. Ask why if Yes/No: _________________

100. Have you *ever* given an animal a shorter duration of antibiotic treatment than recommended on the label (or product leaflet)?

- ◻ Yes
- ◻ No
- ◻ Don’t know

101. Ask why if Yes/No: _________________

102. Have you *ever* given farmers antibiotics to inject animals themselves? [Tick only one]

- ◻ Yes
- ◻ No
- ◻ Don’t know

103. Do you know of farmers who do not speak with a livestock or vet technician before using antibiotics in animals?

- ◻ Yes
- ◻ No

104. If yes, then do you know why they do this

____
____)

105. Have you *ever* advised farmers to give antibiotics to animals without first examining the animals?

- ◻ Yes
- ◻ No
- ◻ Don't know

106. How do you store antibiotics in the office? [Tick all relevant] [Read out]

- ◻ Under direct sunlight
- ◻ Room with air-condition
- ◻ Room without air-condition
- ◻ Refrigerated
- ◻ Others: ____
- ◻ I do not use antibiotics in animals

107. How do you transport antibiotics while on the way to farm? [Tick all relevant]

- ◻ Normal bag
- ◻ Cool-box
- ◻ Others: ____
- ◻ I do not use antibiotics in animals

108. Do you record antibiotic that you have used on "vijilansia" forms? (Select only one)

- ◻ Always
- ◻ Most of the time
- ◻ Sometimes
- ◻ Never
- ◻ I do not use antibiotics in animals

109. Do you check expiry date of antibiotic before using? (Select only one)

- ◻ Always
- ◻ Most of the time
- ◻ Sometimes
- ◻ Never
- ◻ I do not use antibiotics in animals

110. Do you advice farmers to wait for a few days after giving antibiotics before selling, slaughtering or eating products from their animals? (Select only one)

- ◻ Always
- ◻ Most of the time
- ◻ Sometimes
- ◻ Never
- ◻ I do not use antibiotics in animals

111. What else do you tell farmers before or after giving antibiotics? (Tick all that are relevant) [Don't read out but give time to respond]

- ◻ Impact of antimicrobial resistance
- ◻ Possibility that animal might not improve
- ◻ Antibiotics were not used according to the label (off-label use)
- ◻ Storage condition (if antibiotic is given for farmer to self-administer)
- ◻ Duration of treatment required
- ◻ Adverse effects
- ◻ Importance of diagnostic testing
- ◻ Others: ____
- ◻ Nothing
- ◻ I do not use antibiotics in animals

Section 4 – Attitudes [New]

"Attitudes are determined as level of sensitivity to the risks of antimicrobial use; and the appropriate use of antibiotics and alternatives (vaccines/biosecurity)".

[Internal note: The response that indicates a lower sensitivity to the risk of antimicrobial use is highlighted in yellow]

112. I believe giving sick animal *2 or more* types of antibiotics at the same time is *always* better than one.

- ☐ Yes
- ☐ No
- ☐ Don't know

113. I believe that the more antibiotics are used, the less likely they will work in the future.

- ☐ Yes
- ☐ No
- ☐ Don't know

114. I believe skipping 1 or 2 doses of antibiotics can lead to antibiotics becoming less effective in the long term

- ☐ Yes
- ☐ No
- ☐ Don't know

115. I believe that using broad spectrum antibiotics is *always* a better choice than narrow spectrum antibiotics

- ☐ Yes
- ☐ No
- ☐ Don't know

116. I believe using expired antibiotics *can* lead to antibiotics becoming less effective in the long term

- ☐ Yes
- ☐ No
- ☐ Don't know

117. I believe that it is appropriate to give antibiotics for a shorter duration than recommended on the drug label if a sick animal is recovering.

- ◻ Yes
- ◻ No
- ◻ Don’t know

118. I believe that laboratory results can help veterinary and livestock technicians make decisions on antibiotic use in animals?”

- ◻ Yes
- ◻ No
- ◻ Don’t know

119. I believe in giving *healthy* animals antibiotics to help them grow faster

- ◻ Yes
- ◻ No
- ◻ Don’t know

120. I believe that use of vaccines can reduce the use of antibiotics

- ◻ Yes
- ◻ No
- ◻ Don’t know

121. I believe that implementing good farm biosecurity can reduce use of antibiotics

- ◻ Yes
- ◻ No
- ◻ Don’t know

122. I believe that having good animal husbandry and hygienic practice can reduce use of antibiotics?

- ◻ Yes
- ◻ No
- ◻ Don’t know
