# Supplementary material for: Knowledge, attitudes and practices of government animal health workers on antibiotic use and antibiotic resistance in Timor-Leste
Source: Front Vet Sci. 2022 Nov 24;9:1063530. doi: 10.3389/fvets.2022.1063530 (PMC9731573; doi:10.3389/fvets.2022.1063530)
Supplement: Supplementary file 3 [file Data_Sheet_2.docx]

**Supplementary Table 1: Absolute and relative frequency of categorical demographic variables by position and overall**

| Attribute | Veterinary Technicians  (n=57) | Livestock Technicians  (n=87) | Other  Positions  (n=22) | Overall  (n=166) |
| --- | --- | --- | --- | --- |
| Municipality |  |  |  |  |
| Aileu | 3 (5.3) | 4 (4.6) | 3 (13.6) | 10 (6.0) |
| Ainaro | 3 (5.3) | 5 (5.7) | 3 (13.6) | 11 (6.6) |
| Baucau | 4 (7.0) | 6 (6.9) | 1 (4.5) | 11 (6.6) |
| Bobonaro | 9 (15.8) | 9 (10.3) | 0 (0.0) | 18 (10.8) |
| Covalima | 3 (5.3) | 9 (10.3) | 3 (13.6) | 15 (9.0) |
| Dili | 8 (14.0) | 4 (4.6) | 0 (0.0) | 12 (7.2) |
| Ermera | 2 (3.5) | 6 (6.9) | 7 (31.8) | 15 (9.0) |
| Lautem | 5 (8.8) | 9 (10.3) | 0 (0.0) | 14 (8.4) |
| Liquica | 3 (5.3) | 4 (4.6) | 2 (9.1) | 9 (5.4) |
| Manatuto | 5 (8.8) | 7 (8.0) | 0 (0.0) | 12 (7.2) |
| Manufahi | 5 (8.8) | 5 (5.7) | 2 (9.1) | 12 (7.2) |
| Oecusse | 4 (7.0) | 10 (11.5) | 0 (0.0) | 14 (8.4) |
| Viqueque | 3 (5.3) | 9 (10.3) | 1 (4.5) | 13 (7.8) |
| Gender |  |  |  |  |
| Male | 40 (70.2) | 85 (97.7) | 20 (90.9) | 145 (87.3) |
| Female | 17 (29.8) | 2 (2.3) | 2 (9.1) | 21 (12.7) |
| Age (years) |  |  |  |  |
| < 30 | 10 (17.5) | 0 (0.0) | 3 (13.6) | 13 (7.8) |
| 30 - 39 | 43 (75.4) | 16 (18.4) | 5 (22.7) | 64 (38.6) |
| 40 - 49 | 2 (3.5) | 40 (46.0) | 8 (36.4) | 50 (30.1) |
| ≥ 50 | 2 (3.5) | 31 (35.6) | 6 (27.3) | 39 (23.5) |
| Highest education |  |  |  |  |
| University - animal science | 2 (3.5) | 13 (14.9) | 2 (9.1) | 17 (10.2) |
| University - animal health | 53 (93.0) | 0 (0.0) | 1 (4.5) | 54 (32.5) |
| University - other | 0 (0.0) | 4 (4.6) | 7 (31.8) | 11 (6.6) |
| Senior high school - agriculture | 2 (3.5) | 53 (60.9) | 11 (50.0) | 66 (39.8) |
| Senior high school - other | 0 (0.0) | 13 (14.9) | 1 (4.5) | 14 (8.4) |
| Junior high school | 0 (0.0) | 3 (3.4) | 0 (0.0) | 3 (1.8) |
| Others | 0 (0.0) | 1 (1.1) | 0 (0.0) | 1 (0.6) |
| Highest education country |  |  |  |  |
| Timor-Leste | 56 (98.2) | 74 (85.1) | 22 (100.0) | 152 (91.6) |
| Indonesia | 1 (1.8) | 13 (14.9) | 0 (0.0) | 14 (8.4) |
| Work location |  |  |  |  |
| Home | 2 (3.5) | 11 (12.6) | 13 (59.1) | 26 (15.7) |
| Municipal office | 26 (45.6) | 28 (32.2) | 5 (22.7) | 59 (35.5) |
| Posto Administrativo office | 12 (21.1) | 28 (32.2) | 2 (9.1) | 42 (25.3) |
| Animal Health Centre | 11 (19.3) | 8 (9.2) | 1 (4.5) | 20 (12.0) |
| National Directorate of Veterinary | 5 (8.8) | 2 (2.3) | 0 (0.0) | 7 (4.2) |
| Animal Production Centre | 0 (0.0) | 6 (6.9) | 0 (0.0) | 6 (3.6) |
| Extension Centre | 1 (1.8) | 4 (4.6) | 1 (4.5) | 6 (3.6) |
| Assigned to Posto Administrativo(s) |  |  |  |  |
| Yes | 39 (68.4) | 62 (71.3) | 20 (90.9) | 121 (72.9) |
| No | 18 (31.6) | 25 (28.7) | 2 (9.1) | 45 (27.1) |
| Purpose of farm visit^1^ |  |  |  |  |
| Vaccination | 56 (98.2) | 84 (96.6) | 19 (86.4) | 159 (95.8) |
| Treatment of sick animals | 57 (100.0) | 86 (98.9) | 22 (100.0) | 165 (99.4) |
| Advice on livestock disease | 9 (15.8) | 11 (12.6) | 2 (9.1) | 22 (13.3) |
| Advice on livestock management | 9 (15.8) | 17 (19.5) | 1 (4.5) | 27 (16.3) |
| Field project implementation | 7 (12.3) | 10 (11.5) | 0 (0.0) | 17 (10.2) |
| Animal health data collection | 1 (1.8) | 2 (2.3) | 2 (9.1) | 5 (3.0) |
| Other | 5 (8.8) | 2 (2.3) | 0 (0.0) | 7 (4.2) |
| Know send samples to lab |  |  |  |  |
| Yes | 57 (100.0) | 86 (98.9) | 21 (95.5) | 164 (98.8) |
| No | 0 (0.0) | 1 (1.1) | 1 (4.5) | 2 (1.2) |
| Ever sent samples to lab |  |  |  |  |
| Yes | 43 (75.4) | 65 (74.7) | 9 (40.9) | 117 (70.5) |
| No | 14 (24.6) | 21 (24.1) | 12 (54.5) | 47 (28.3) |
| Number of samples from sick animals sent to lab in last 12 months^2^ | | |  |  |
| 1 - 5 | 27 (62.8) | 49 (75.4) | 9 (100.0) | 85 (72.6) |
| 6 - 10 | 2 (4.7) | 5 (7.7) | 0 (0.0) | 7 (6.0) |
| 11 - 20 | 2 (4.7) | 3 (4.6) | 0 (0.0) | 5 (4.3) |
| > 20 | 11 (25.6) | 7 (10.8) | 0 (0.0) | 18 (15.4) |
| Not stated | 1 (2.3) | 1 (1.5) | 0 (0.0) | 2 (1.7) |
| How often lab test received^2^ |  |  |  |  |
| Always | 3 (7.0) | 2 (3.1) | 0 (0.0) | 5 (4.3) |
| Sometimes | 15 (34.9) | 13 (20.0) | 2 (22.2) | 30 (25.6) |
| Never | 25 (58.1) | 50 (76.9) | 7 (77.8) | 82 (70.1) |

^1^Multiple responses allowed

^2^Of those who have previously sent samples to the laboratory (n = 117)

**Supplementary Table 2: Median and interquartile range of continuous demographic variables by position and overall (n = 166)**

|  | Veterinary Technicians | Livestock Technicians | Other Positions | Overall |
| --- | --- | --- | --- | --- |
| Age | 32 (30 - 33) | 48 (42 - 52) | 46.5 (35.5 - 50) | 41.5 (33 - 49) |
| Years working in the field | 7 ( 6 - 8) | 9 (9 - 13) | 12 (11 - 13) | 9 (8 - 11.25) |
| Average number of farms visited per month | 15 (7.5 - 23.5) | 15 (8 - 20) | 5 (3.75 - 10) | 12 (5 - 20) |

**Supplementary Table 3: Absolute and relative frequency of farm observation variables by position and overall**

| Attribute | | Veterinary Technicians  (n=57) | Livestock Technicians  (n=87) | Other  Positions  (n=22) | Overall  (n=166) |
| --- | --- | --- | --- | --- | --- |
| Percentage chickens thought to be vaccinated against ND in work area | | | |  |  |
| 0 - 33% | 14 (24.6) | | 16 (18.4) | 8 (36.4) | 38 (22.9) |
| 34 - 66% | 27 (47.4) | | 41 (47.1) | 6 (27.3) | 74 (44.6) |
| 67 - 100% | 11 (19.3) | | 26 (29.9) | 4 (18.2) | 41 (24.7) |
| Don't know | 5 (8.8) | | 4 (4.6) | 4 (18.2) | 13 (7.8) |
| Percentage pigs thought to be vaccinated against CSF in work area | | | |  |  |
| 0 - 33% | 36 (63.2) | | 54 (62.1) | 10 (45.5) | 100 (60.2) |
| 34 - 66% | 13 (22.8) | | 25 (28.7) | 8 (36.4) | 46 (27.7) |
| 67 - 100% | 3 (5.3) | | 5 (5.7) | 0 (0.0) | 8 (4.8) |
| Don't know | 5 (8.8) | | 3 (3.4) | 4 (18.2) | 12 (7.2) |
| Observed fencing around visited farms | | |  |  |  |
| Never | 16 (28.1) | | 30 (34.5) | 11 (50.0) | 57 (34.3) |
| Occasionally | 40 (70.2) | | 56 (64.4) | 11 (50.0) | 107 (64.5) |
| Frequently | 1 (1.8) | | 1 (1.1) | 0 (0.0) | 2 (1.2) |
| Observed locked gate on visited farms | | |  |  |  |
| Never | 22 (38.6) | | 53 (60.9) | 14 (63.6) | 89 (53.6) |
| Occasionally | 35 (61.4) | | 33 (37.9) | 8 (36.4) | 76 (45.8) |
| Frequently | 0 (0.0) | | 1 (1.1) | 0 (0.0) | 1 (0.6) |
| Observed chickens in cages in visited farms | | |  |  |  |
| Never | 7 (12.3) | | 20 (23.0) | 3 (13.6) | 30 (18.1) |
| Occasionally | 48 (84.2) | | 65 (74.7) | 19 (86.4) | 132 (79.5) |
| Frequently | 2 (3.5) | | 2 (2.3) | 0 (0.0) | 4 (2.4) |
| Observed other animals in pens in visited farms | | |  |  |  |
| Never | 24 (42.1) | | 28 (32.2) | 7 (31.8) | 59 (35.5) |
| Occasionally | 32 (56.1) | | 58 (66.7) | 15 (68.2) | 105 (63.3) |
| Frequently | 1 (1.8) | | 1 (1.1) | 0 (0.0) | 2 (1.2) |
| Observed farmers wearing dedicated boots when tending to animals on visited farms | | | | |  |
| Never | 42 (73.7) | | 67 (77.0) | 17 (77.3) | 126 (75.9) |
| Occasionally | 15 (26.3) | | 20 (23.0) | 5 (22.7) | 40 (24.1) |
| Frequently | 0 (0.0) | | 0 (0.0) | 0 (0.0) | 0 (0.0) |
| Observed disinfection of footwear during visits to farms | | | |  |  |
| Never | 41 (71.9) | | 73 (83.9) | 19 (86.4) | 133 (80.1) |
| Occasionally | 15 (26.3) | | 13 (14.9) | 3 (13.6) | 31 (18.7) |
| Frequently | 1 (1.8) | | 1 (1.1) | 0 (0.0) | 2 (1.2) |
| Observed a "sign-in" during visits to farms | | |  |  |  |
| Never | 47 (82.5) | | 71 (81.6) | 18 (81.8) | 136 (81.9) |
| Occasionally | 9 (15.8) | | 16 (18.4) | 4 (18.2) | 29 (17.5) |
| Frequently | 1 (1.8) | | 0 (0.0) | 0 (0.0) | 1 (0.6) |

**Supplementary Table 4: Absolute and relative frequency of responses on knowledge of antibiotics by position and overall**

| Attribute | Veterinary Technicians  (n=56) | Livestock Technicians  (n=85) | Other Positions  (n=21) | Overall  (n=162) |
| --- | --- | --- | --- | --- |
| Know what antibiotics are |  |  |  |  |
| Yes | 56 (100.0) | 85 (100.0) | 21 (100.0) | 162 (100.0) |
| No | 0 (0.0) | 0 (0.0) | 0 (0.0) | 0 (0.0) |
| Where learnt about antibiotics | |  |  |  |
| University | 47 (83.9) | 10 (11.8) | 3 (14.3) | 60 (37.0) |
| Senior high school | 7 (12.5) | 36 (42.4) | 7 (33.3) | 50 (30.9) |
| At work | 5 (8.9) | 10 (11.8) | 4 (19.0) | 19 (11.7) |
| MAF training course | 23 (41.1) | 68 (80.0) | 16 (76.2) | 107 (66.0) |
| Non-MAF training course | 2 (3.6) | 1 (1.2) | 0 (0.0) | 3 (1.9) |
| Other | 0 (0.0) | 1 (1.2) | 0 (0.0) | 1 (0.6) |
| Antibiotic kills bacteria |  |  |  |  |
| Correct | 49 (87.5) | 76 (89.4) | 17 (81.0) | 142 (87.7) |
| Incorrect | 5 (8.9) | 4 (4.7) | 2 (9.5) | 11 (6.8) |
| Don't know | 2 (3.6) | 5 (5.9) | 2 (9.5) | 9 (5.6) |
| Antibiotic kills virus |  |  |  |  |
| Correct | 43 (76.8) | 46 (54.1) | 8 (38.1) | 97 (59.9) |
| Incorrect | 11 (19.6) | 31 (36.5) | 11 (52.4) | 53 (32.7) |
| Don't know | 2 (3.6) | 8 (9.4) | 2 (9.5) | 12 (7.4) |
| Antibiotic kills parasite |  |  |  |  |
| Correct | 41 (73.2) | 40 (47.1) | 7 (33.3) | 88 (54.3) |
| Incorrect | 12 (21.4) | 37 (43.5) | 9 (42.9) | 58 (35.8) |
| Don't know | 3 (5.4) | 8 (9.4) | 5 (23.8) | 16 (9.9) |
| Antibiotic directly reduces inflammation |  |  |  |  |
| Correct | 8 (14.3) | 3 (3.5) | 0 (0.0) | 11 (6.8) |
| Incorrect | 46 (82.1) | 76 (89.4) | 19 (90.5) | 141 (87.0) |
| Don't know | 2 (3.6) | 6 (7.1) | 2 (9.5) | 10 (6.2) |
| Antibiotic directly reduces fever | |  |  |  |
| Correct | 10 (17.9) | 6 (7.1) | 0 (0.0) | 16 (9.9) |
| Incorrect | 45 (80.4) | 76 (89.4) | 19 (90.5) | 140 (86.4) |
| Don't know | 1 (1.8) | 3 (3.5) | 2 (9.5) | 6 (3.7) |
| Four answers correct | 9 (16.1) | 4 (4.7) | 0 (0.0) | 13 (8.0) |
| Bacteria and virus correct | 40 (71.4) | 45 (52.9) | 8 (38.1) | 93 (57.4) |
| Identified Medoxy-LA as an antibiotic | |  |  |  |
| Correct | 55 (98.2) | 84 (98.8) | 19 (90.5) | 158 (97.5) |
| Incorrect | 1 (1.8) | 1 (1.2) | 2 (9.5) | 4 (2.5) |
| Don't know | 0 (0.0) | 0 (0.0) | 0 (0.0) | 0 (0.0) |
| Identified Vitamin B as not an antibiotic | |  |  |  |
| Correct | 50 (89.3) | 72 (84.7) | 20 (95.2) | 142 (87.7) |
| Incorrect | 5 (8.9) | 12 (14.1) | 1 (4.8) | 18 (11.1) |
| Don't know | 1 (1.8) | 1 (1.2) | 0 (0.0) | 2 (1.2) |
| Identified Ivermectin as not an antibiotic | |  |  |  |
| Correct | 45 (80.4) | 43 (50.6) | 7 (33.3) | 95 (58.6) |
| Incorrect | 11 (19.6) | 42 (49.4) | 14 (66.7) | 67 (41.4) |
| Don't know | 0 (0.0) | 0 (0.0) | 0 (0.0) | 0 (0.0) |
| Heard about broad and narrow spectrum antibiotics | | |  |  |
| Yes | 48 (85.7) | 54 (63.5) | 11 (52.4) | 113 (69.8) |
| No | 8 (14.3) | 31 (36.5) | 10 (47.6) | 49 (30.2) |
| Identified Medoxy-LA as broad spectrum antibiotic^1^ | | |  |  |
| Correct | 43 (89.6) | 47 (87.0) | 6 (54.5) | 96 (85.0) |
| Incorrect | 1 (2.1) | 4 (7.4) | 3 (27.3) | 8 (7.1) |
| Don't know | 4 (8.3) | 3 (5.6) | 2 (18.2) | 9 (8.0) |
| Heard of critically important antimicrobial for human medicine | | | |  |
| Yes | 21 (37.5) | 32 (37.6) | 11 (52.4) | 64 (39.5) |
| No | 35 (62.5) | 53 (62.4) | 10 (47.6) | 98 (60.5) |

^1^Of those who had heard of broad and narrow spectrum antibiotics (n = 113)

**Supplementary Table 5: Absolute and relative frequency of responses to attitude statements by position and overall. “Correct” responses are shaded in grey.**

| Attitude statement | Veterinary Technicians  (n=56) | Livestock Technicians  (n=85) | Other Positions  (n=21) | Overall  (n=162) |
| --- | --- | --- | --- | --- |
| I believe giving sick animal 2 or more types of antibiotics at the same time is always better than one. | | | | |
| Yes | 17 (30.4) | 48 (56.5) | 8 (38.1) | 73 (45.1) |
| No | 33 (58.9) | 26 (30.6) | 9 (42.9) | 68 (42.0) |
| Don't know | 6 (10.7) | 11 (12.9) | 4 (19.0) | 21 (13.0) |
| I believe that the more antibiotics are used, the less likely they will work in the future | | | | |
| Yes | 39 (69.6) | 49 (57.6) | 9 (42.9) | 97 (59.9) |
| No | 11 (19.6) | 26 (30.6) | 8 (38.1) | 45 (27.8) |
| Don't know | 6 (10.7) | 10 (11.8) | 4 (19.0) | 20 (12.3) |
| I believe skipping 1 or 2 doses of antibiotics can lead to antibiotics becoming less effective in the long term | | | | |
| Yes | 34 (60.7) | 49 (57.6) | 13 (61.9) | 96 (59.3) |
| No | 16 (28.6) | 19 (22.4) | 5 (23.8) | 40 (24.7) |
| Don't know | 6 (10.7) | 17 (20.0) | 3 (14.3) | 26 (16.0) |
| I believe that using broad spectrum antibiotics is always a better choice than narrow spectrum antibiotics | | | | |
| Yes | 48 (85.7) | 62 (72.9) | 17 (81.0) | 127 (78.4) |
| No | 3 (5.4) | 12 (14.1) | 0 (0.0) | 15 (9.3) |
| Don't know | 5 (8.9) | 11 (12.9) | 4 (19.0) | 20 (12.3) |
| I believe using expired antibiotics can lead to antibiotics becoming less effective in the long term | | | | |
| Yes | 47 (83.9) | 66 (77.6) | 16 (76.2) | 129 (79.6) |
| No | 8 (14.3) | 16 (18.8) | 5 (23.8) | 29 (17.9) |
| Don't know | 1 (1.8) | 3 (3.5) | 0 (0.0) | 4 (2.5) |
| I believe that it is appropriate to give antibiotics for a shorter duration than recommended on the drug label if a sick animal is recovering | | | | |
| Yes | 36 (64.3) | 60 (70.6) | 12 (57.1) | 108 (66.7) |
| No | 18 (32.1) | 15 (17.6) | 6 (28.6) | 39 (24.1) |
| Don't know | 2 (3.6) | 10 (11.8) | 3 (14.3) | 15 (9.3) |
| I believe that laboratory results can help veterinary and livestock technicians make decisions on antibiotic use in animals?” | | | | |
| Yes | 54 (96.4) | 79 (92.9) | 21 (100.0) | 154 (95.1) |
| No | 1 (1.8) | 2 (2.4) | 0 (0.0) | 3 (1.9) |
| Don't know | 1 (1.8) | 4 (4.7) | 0 (0.0) | 5 (3.1) |
| I believe in giving healthy animals antibiotics to help them grow | | | | |
| Yes | 6 (10.7) | 23 (27.1) | 3 (14.3) | 32 (19.8) |
| No | 45 (80.4) | 51 (60.0) | 15 (71.4) | 111 (68.5) |
| Don't know | 5 (8.9) | 11 (12.9) | 3 (14.3) | 19 (11.7) |
| I believe that use of vaccines can reduce the use of antibiotics | | | | |
| Yes | 49 (87.5) | 65 (76.5) | 15 (71.4) | 129 (79.6) |
| No | 7 (12.5) | 15 (17.6) | 4 (19.0) | 26 (16.0) |
| Don't know | 0 (0.0) | 5 (5.9) | 2 (9.5) | 7 (4.3) |
| I believe that implementing good farm biosecurity can reduce use of antibiotics | | | | |
| Yes | 53 (94.6) | 75 (88.2) | 21 (100.0) | 149 (92.0) |
| No | 2 (3.6) | 5 (5.9) | 0 (0.0) | 7 (4.3) |
| Don't know | 1 (1.8) | 5 (5.9) | 0 (0.0) | 6 (3.7) |
| I believe that having good animal husbandry and hygienic practice can reduce use of antibiotics? | | | | |
| Yes | 53 (94.6) | 82 (96.5) | 21 (100.0) | 156 (96.3) |
| No | 2 (3.6) | 1 (1.2) | 0 (0.0) | 3 (1.9) |
| Don't know | 1 (1.8) | 2 (2.4) | 0 (0.0) | 3 (1.9) |

**Supplementary Table 6: Absolute and relative frequency of antibiotic use practices by position and overall**

| Attribute | Veterinary Technicians  (n=56) | Livestock Technicians  (n=85) | Other Positions  (n=21) | Overall  (n=162) |
| --- | --- | --- | --- | --- |
| Ever used antibiotics for work |  |  |  |  |
| Yes | 56 (100.0) | 85 (100.0) | 21 (100.0) | 162 (100.0) |
| No | 0 (0.0) | 0 (0.0) | 0 (0.0) | 0 (0.0) |
| Don't know | 0 (0.0) | 0 (0.0) | 0 (0.0) | 0 (0.0) |
| Average number of animals treated with antibiotics per month prior to COVID-19 | | |  |  |
| 0 - 5 | 10 (17.9) | 13 (15.3) | 9 (42.9) | 32 (19.8) |
| 6 - 10 | 12 (21.4) | 20 (23.5) | 7 (33.3) | 39 (24.1) |
| 11 - 15 | 8 (14.3) | 12 (14.1) | 1 (4.8) | 21 (13.0) |
| 16 - 20 | 11 (19.6) | 20 (23.5) | 2 (9.5) | 33 (20.4) |
| > 20 | 15 (26.8) | 20 (23.5) | 2 (9.5) | 37 (22.8) |
| Used antibiotics in sick animals to help them recover | |  |  |  |
| Yes | 56 (100.0) | 85 (100.0) | 20 (95.2) | 161 (99.4) |
| No | 0 (0.0) | 0 (0.0) | 0 (0.0) | 0 (0.0) |
| Don't know | 0 (0.0) | 0 (0.0) | 1 (4.8) | 1 (0.6) |
| Used antibiotics in healthy animals in contact with sick animals | |  |  |  |
| Yes | 26 (46.4) | 45 (52.9) | 9 (42.9) | 80 (49.4) |
| No | 30 (53.6) | 40 (47.1) | 12 (57.1) | 82 (50.6) |
| Used antibiotics to help healthy animals grow faster | |  |  |  |
| Yes | 1 (1.8) | 5 (5.9) | 0 (0.0) | 6 (3.7) |
| No | 55 (98.2) | 80 (94.1) | 21 (100.0) | 156 (96.3) |
| What other options do you use for treating sick animals | |  |  |  |
| Anti-inflammatory | 12 (21.4) | 10 (11.8) | 1 (4.8) | 23 (14.2) |
| Vitamin B complex | 54 (96.4) | 83 (97.6) | 19 (90.5) | 156 (96.3) |
| Herbal medicine | 0 (0.0) | 4 (4.7) | 3 (14.3) | 7 (4.3) |
| Anti-parasitics/anti-protozoal | 39 (69.6) | 56 (65.9) | 11 (52.4) | 106 (65.4) |
| Others | 1 (1.8) | 1 (1.2) | 0 (0.0) | 2 (1.2) |
| None | 2 (3.6) | 1 (1.2) | 1 (4.8) | 4 (2.5) |
| Always give antibiotics to sick animals |  |  |  |  |
| Yes | 48 (85.7) | 67 (78.8) | 18 (85.7) | 133 (82.1) |
| No | 8 (14.3) | 18 (21.2) | 3 (14.3) | 29 (17.9) |
| If no, why not used antibiotics^1^ |  |  |  |  |
| No antibiotics available | 0 (0.0) | 2 (11.1) | 3 (100.0) | 5 (17.2) |
| No appropriate antibiotic available | 1 (12.5) | 0 (0.0) | 0 (0.0) | 1 (3.4) |
| Conserving antibiotics because supply is low | 0 (0.0) | 0 (0.0) | 0 (0.0) | 0 (0.0) |
| Moribund animal | 0 (0.0) | 0 (0.0) | 0 (0.0) | 0 (0.0) |
| Low value animal | 0 (0.0) | 0 (0.0) | 0 (0.0) | 0 (0.0) |
| Animal unlikely to have bacterial infection | 7 (87.5) | 15 (83.3) | 0 (0.0) | 22 (75.9) |
| Do not want to be blamed by farmer for death of animal | 0 (0.0) | 0 (0.0) | 0 (0.0) | 0 (0.0) |
| Residues in meat if animal dies before end of WHP | 0 (0.0) | 0 (0.0) | 0 (0.0) | 0 (0.0) |
| Other | 0 (0.0) | 1 (5.6) | 0 (0.0) | 1 (3.4) |
| If no, faced resistance from farmer for deciding not to use antibiotics in sick animal^1^ | | |  |  |
| Yes | 4 (50.0) | 2 (11.1) | 1 (33.3) | 7 (24.1) |
| No | 4 (50.0) | 16 (88.9) | 2 (66.7) | 22 (75.9) |

^1^Of those who did not always give antibiotics to sick animals (n = 29)

**Supplementary Table 7: Absolute and relative frequency of reports of lack of supply and purchase of antibiotics by position and overall**

| Attribute | Veterinary Technicians  (n=56) | Livestock Technicians  (n=85) | Other Positions  (n=21) | Overall  (n=162) |
| --- | --- | --- | --- | --- |
| Lacking in supply |  |  |  |  |
| Medoxy-LA |  |  |  |  |
| Yes | 46 (82.1) | 72 (84.7) | 19 (90.5) | 137 (84.6) |
| No | 10 (17.9) | 10 (11.8) | 2 (9.5) | 22 (13.6) |
| Don't know | 0 (0.0) | 3 (3.5) | 0 (0.0) | 3 (1.9) |
| Ivermectin |  |  |  |  |
| Yes | 45 (80.4) | 65 (76.5) | 20 (95.2) | 130 (80.2) |
| No | 11 (19.6) | 17 (20.0) | 1 (4.8) | 29 (17.9) |
| Don't know | 0 (0.0) | 3 (3.5) | 0 (0.0) | 3 (1.9) |
| Albendissu |  |  |  |  |
| Yes | 41 (73.2) | 63 (74.1) | 14 (66.7) | 118 (72.8) |
| No | 13 (23.2) | 19 (22.4) | 4 (19.0) | 36 (22.2) |
| Don't know | 2 (3.6) | 3 (3.5) | 3 (14.3) | 8 (4.9) |
| Pen-strep 400 |  |  |  |  |
| Yes | 32 (57.1) | 64 (75.3) | 16 (76.2) | 112 (69.1) |
| No | 23 (41.1) | 17 (20.0) | 2 (9.5) | 42 (25.9) |
| Don't know | 1 (1.8) | 4 (4.7) | 3 (14.3) | 8 (4.9) |
| Sulfa strong(sulfabac) |  |  |  |  |
| Yes | 37 (66.1) | 66 (77.6) | 13 (61.9) | 116 (71.6) |
| No | 18 (32.1) | 10 (11.8) | 3 (14.3) | 31 (19.1) |
| Don't know | 1 (1.8) | 9 (10.6) | 5 (23.8) | 15 (9.3) |
| Colibact |  |  |  |  |
| Yes | 26 (46.4) | 31 (36.5) | 4 (19.0) | 61 (37.7) |
| No | 12 (21.4) | 17 (20.0) | 1 (4.8) | 30 (18.5) |
| Don't know | 18 (32.1) | 37 (43.5) | 16 (76.2) | 71 (43.8) |
| Gusanex |  |  |  |  |
| Yes | 43 (76.8) | 75 (88.2) | 20 (95.2) | 138 (85.2) |
| No | 12 (21.4) | 7 (8.2) | 1 (4.8) | 20 (12.3) |
| Don't know | 1 (1.8) | 3 (3.5) | 0 (0.0) | 4 (2.5) |
| Intramox |  |  |  |  |
| Yes | 17 (30.4) | 31 (36.5) | 6 (28.6) | 54 (33.3) |
| No | 20 (35.7) | 15 (17.6) | 2 (9.5) | 37 (22.8) |
| Don't know | 19 (33.9) | 39 (45.9) | 13 (61.9) | 71 (43.8) |
| Trypamidium |  |  |  |  |
| Yes | 15 (26.8) | 17 (20.0) | 4 (19.0) | 36 (22.2) |
| No | 17 (30.4) | 13 (15.3) | 0 (0.0) | 30 (18.5) |
| Don't know | 24 (42.9) | 55 (64.7) | 17 (81.0) | 96 (59.3) |
| Oxytetracline HCL |  |  |  |  |
| Yes | 34 (60.7) | 69 (81.2) | 17 (81.0) | 120 (74.1) |
| No | 21 (37.5) | 13 (15.3) | 2 (9.5) | 36 (22.2) |
| Don't know | 1 (1.8) | 3 (3.5) | 2 (9.5) | 6 (3.7) |
| Ever obtained antibiotics for work from sources other than MAF | | | |  |
| Yes | 12 (21.4) | 23 (27.1) | 1 (4.8) | 36 (22.2) |
| No | 44 (78.6) | 62 (72.9) | 20 (95.2) | 126 (77.8) |
| Which sources^1^ |  |  |  |  |
| Agriculture shop | 10 (83.3) | 18 (78.3) | 1 (100.0) | 29 (80.6) |
| Agriculture shop - purchased by farmer | 0 (0.0) | 1 (4.3) | 0 (0.0) | 1 (2.8) |
| Agriculture shop - in Indonesia | 0 (0.0) | 2 (8.7) | 0 (0.0) | 2 (5.6) |
| Market vendor | 0 (0.0) | 1 (4.3) | 0 (0.0) | 1 (2.8) |
| Pharmacy | 1 (8.3) | 0 (0.0) | 0 (0.0) | 1 (2.8) |
| UNTL | 1 (8.3) | 0 (0.0) | 0 (0.0) | 1 (2.8) |
| Australian veterinarian who visited Timor-Leste | 0 (0.0) | 1 (4.3) | 0 (0.0) | 1 (2.8) |

^1^Of those who had obtained antibiotics for work from sources other than MAF (n = 36)

**Supplementary Table 8: Absolute and relative frequency of decisions on choice of antibiotic by position and overall**

| Decision | Veterinary Technicians  (n=56) | Livestock Technicians  (n=85) | Other Positions  (n=21) | Overall  (n=162) |
| --- | --- | --- | --- | --- |
| I decide on which antibiotic to use in a sick animal based on my past experience on what has worked for the suspected disease | | | | |
| Always | 50 (89.3) | 83 (97.6) | 19 (90.5) | 152 (93.8) |
| Sometimes | 6 (10.7) | 2 (2.4) | 2 (9.5) | 10 (6.2) |
| Never | 0 (0.0) | 0 (0.0) | 0 (0.0) | 0 (0.0) |
| I decide on which antibiotic to use in a sick animal based on my colleague's experience on what has worked for the suspected disease | | | | |
| Always | 9 (16.1) | 38 (44.7) | 9 (42.9) | 56 (34.6) |
| Sometimes | 26 (46.4) | 24 (28.2) | 8 (38.1) | 58 (35.8) |
| Never | 21 (37.5) | 23 (27.1) | 4 (19.0) | 48 (29.6) |
| I decide on which antibiotic to use in a sick animal based on what I learnt at university or high school | | | | |
| Always | 49 (87.5) | 58 (68.2) | 14 (66.7) | 121 (74.7) |
| Sometimes | 7 (12.5) | 9 (10.6) | 3 (14.3) | 19 (11.7) |
| Never | 0 (0.0) | 18 (21.2) | 4 (19.0) | 22 (13.6) |
| I decide on which antibiotic to use in a sick animal based on what I learnt at a post-graduation training course | | | | |
| Always | 43 (76.8) | 66 (77.6) | 15 (71.4) | 124 (76.5) |
| Sometimes | 6 (10.7) | 18 (21.2) | 3 (14.3) | 27 (16.7) |
| Never | 7 (12.5) | 1 (1.2) | 3 (14.3) | 11 (6.8) |
| I decide on which antibiotic to use in a sick animal based on veterinary antibiotic prescribing guidelines from other countries | | | | |
| Always | 0 (0.0) | 2 (2.4) | 0 (0.0) | 2 (1.2) |
| Sometimes | 1 (1.8) | 0 (0.0) | 0 (0.0) | 1 (0.6) |
| Never | 55 (98.2) | 83 (97.6) | 21 (100.0) | 159 (98.1) |
| I decide on which antibiotic to use in a sick animal based on what is available | | | | |
| Always | 51 (91.1) | 77 (90.6) | 17 (81.0) | 145 (89.5) |
| Sometimes | 4 (7.1) | 7 (8.2) | 4 (19.0) | 15 (9.3) |
| Never | 1 (1.8) | 1 (1.2) | 0 (0.0) | 2 (1.2) |
| I decide on which antibiotic to use in a sick animal based on which is cheaper | | | | |
| Always | 0 (0.0) | 1 (1.2) | 1 (4.8) | 2 (1.2) |
| Sometimes | 7 (12.5) | 7 (8.2) | 3 (14.3) | 17 (10.5) |
| Never | 49 (87.5) | 77 (90.6) | 17 (81.0) | 143 (88.3) |
| I decide on which antibiotic to use in a sick animal based on whether the drug label states the antibiotic is effective for the suspected disease | | | | |
| Always | 51 (91.1) | 79 (92.9) | 21 (100.0) | 151 (93.2) |
| Sometimes | 4 (7.1) | 5 (5.9) | 0 (0.0) | 9 (5.6) |
| Never | 1 (1.8) | 1 (1.2) | 0 (0.0) | 2 (1.2) |
| I decide on which antibiotic to use in a sick animal based on duration of action of the antimicrobial | | | | |
| Always | 36 (64.3) | 66 (77.6) | 18 (85.7) | 120 (74.1) |
| Sometimes | 18 (32.1) | 15 (17.6) | 1 (4.8) | 34 (21.0) |
| Never | 2 (3.6) | 4 (4.7) | 2 (9.5) | 8 (4.9) |
| I decide on which antibiotic to use in a sick animal based on whether the meat/milk/eggs can be eaten after giving that antibiotic | | | | |
| Always | 14 (25.0) | 27 (31.8) | 3 (14.3) | 44 (27.2) |
| Sometimes | 22 (39.3) | 32 (37.6) | 6 (28.6) | 60 (37.0) |
| Never | 20 (35.7) | 26 (30.6) | 12 (57.1) | 58 (35.8) |
| I decide on which antibiotic to use in a sick animal based on laboratory test result | | | | |
| Always | 1 (1.8) | 0 (0.0) | 0 (0.0) | 1 (0.6) |
| Sometimes | 1 (1.8) | 7 (8.2) | 0 (0.0) | 8 (4.9) |
| Never | 54 (96.4) | 78 (91.8) | 21 (100.0) | 153 (94.4) |
| I decide on which antibiotic to use in a sick animal based on trial and error | | | | |
| Always | 10 (17.9) | 17 (20.0) | 6 (28.6) | 33 (20.4) |
| Sometimes | 16 (28.6) | 23 (27.1) | 5 (23.8) | 44 (27.2) |
| Never | 30 (53.6) | 45 (52.9) | 10 (47.6) | 85 (52.5) |
| Decide - other criteria | |  |  |  |
| No further criteria or irrelevant response | 30 (53.6) | 43 (50.6) | 10 (47.6) | 83 (51.2) |
| Clinical sign or physical examination findings | 26 (46.4) | 41 (48.2) | 11 (52.4) | 78 (48.1) |
| History findings | 1 (1.8) | 8 (9.4) | 3 (14.3) | 12 (7.4) |

**Supplementary Table 9: Absolute and relative frequency of use of antibiotics by species by position and overall**

| Question | Veterinary Technicians  (n=56) | Livestock Technicians  (n=85) | Other Positions  (n=21) | Overall  (n=162) |
| --- | --- | --- | --- | --- |
| Which species have you ever used antibiotics in (any route of administration)^1^ | | |  |  |
| Local chicken | 41 (73.2) | 48 (56.5) | 11 (52.4) | 100 (61.7) |
| Broiler | 0 (0.0) | 1 (1.2) | 0 (0.0) | 1 (0.6) |
| Layer | 2 (3.6) | 2 (2.4) | 0 (0.0) | 4 (2.5) |
| Fighting cock | 14 (25.0) | 15 (17.6) | 3 (14.3) | 32 (19.8) |
| Pig | 56 (100.0) | 84 (98.8) | 21 (100.0) | 161 (99.4) |
| Cattle | 54 (96.4) | 83 (97.6) | 20 (95.2) | 157 (96.9) |
| Buffalo | 40 (71.4) | 72 (84.7) | 13 (61.9) | 125 (77.2) |
| Goat | 48 (85.7) | 78 (91.8) | 16 (76.2) | 142 (87.7) |
| Dog | 52 (92.9) | 73 (85.9) | 13 (61.9) | 138 (85.2) |
| Cat | 8 (14.3) | 10 (11.8) | 2 (9.5) | 20 (12.3) |
| Horse | 19 (33.9) | 41 (48.2) | 2 (9.5) | 62 (38.3) |
| Deer | 0 (0.0) | 4 (4.7) | 0 (0.0) | 4 (2.5) |
| Monkey | 3 (5.4) | 1 (1.2) | 0 (0.0) | 4 (2.5) |
| Sheep | 2 (3.6) | 1 (1.2) | 1 (4.8) | 4 (2.5) |
| Duck | 0 (0.0) | 1 (1.2) | 0 (0.0) | 1 (0.6) |
| Rabbit | 0 (0.0) | 0 (0.0) | 0 (0.0) | 0 (0.0) |
| Others | 0 (0.0) | 0 (0.0) | 0 (0.0) | 0 (0.0) |
| Top 3 species in which you used antibiotics in last 12 months^1^ | |  |  |  |
| Local chicken | 10 (17.9) | 6 (7.1) | 1 (4.8) | 17 (10.5) |
| Broiler | 0 (0.0) | 0 (0.0) | 0 (0.0) | 0 (0.0) |
| Layer | 0 (0.0) | 1 (1.2) | 0 (0.0) | 1 (0.6) |
| Fighting cock | 3 (5.4) | 0 (0.0) | 0 (0.0) | 3 (1.9) |
| Pig | 50 (89.3) | 70 (82.4) | 18 (85.7) | 138 (85.2) |
| Cattle | 42 (75.0) | 76 (89.4) | 19 (90.5) | 137 (84.6) |
| Buffalo | 12 (21.4) | 52 (61.2) | 9 (42.9) | 73 (45.1) |
| Goat | 27 (48.2) | 35 (41.2) | 10 (47.6) | 72 (44.4) |
| Dog | 23 (41.1) | 13 (15.3) | 6 (28.6) | 42 (25.9) |
| Cat | 0 (0.0) | 0 (0.0) | 0 (0.0) | 0 (0.0) |
| Horses | 1 (1.8) | 3 (3.5) | 0 (0.0) | 4 (2.5) |
| None of the above | 0 (0.0) | 0 (0.0) | 0 (0.0) | 0 (0.0) |
| Which antibiotics have you used in pigs in the last 12 months (any route of administration) ^1,2^ | | |  |  |
| Penstrep | 30 (53.6) | 41 (48.2) | 7 (33.3) | 78 (48.1) |
| Medoxy-LA | 53 (94.6) | 78 (91.8) | 16 (76.2) | 147 (90.7) |
| Sulfa-strong/sulfabac | 18 (32.1) | 18 (21.2) | 8 (38.1) | 44 (27.2) |
| Oxytetracycline (Salep Mata) | 5 (8.9) | 14 (16.5) | 1 (4.8) | 20 (12.3) |
| Don't know | 0 (0.0) | 0 (0.0) | 0 (0.0) | 0 (0.0) |
| Don't use antibiotics in pigs | 0 (0.0) | 1 (1.2) | 0 (0.0) | 1 (0.6) |
| Vet-Oxy LA | 8 (14.3) | 11 (12.9) | 0 (0.0) | 19 (11.7) |
| Oxytetracycline LA | 0 (0.0) | 3 (3.5) | 5 (23.8) | 8 (4.9) |
| Tetra (human) | 0 (0.0) | 1 (1.2) | 0 (0.0) | 1 (0.6) |
| Terramycin | 0 (0.0) | 2 (2.4) | 0 (0.0) | 2 (1.2) |
| Colibact | 1 (1.8) | 0 (0.0) | 0 (0.0) | 1 (0.6) |
| Amoxicillin | 0 (0.0) | 1 (1.2) | 0 (0.0) | 1 (0.6) |
| Listed drug is not an antibiotic | 0 (0.0) | 1 (1.2) | 0 (0.0) | 1 (0.6) |
| What are the classes of drugs used in pigs in the last 12 months? (any route of administration) of those who used antibiotics in pigs^1,2^ | | | | |
| Tetracycline | 55 (98.2) | 82 (97.6) | 20 (95.2) | 157 (97.5) |
| Penicillin | 30 (53.6) | 41 (48.8) | 7 (33.3) | 78 (48.4) |
| Aminoglycoside | 30 (53.6) | 41 (48.8) | 7 (33.3) | 78 (48.4) |
| Sulfonamide | 19 (33.9) | 18 (21.4) | 8 (38.1) | 45 (28.0) |
| Was the antibiotics used in pigs in the last 12 months a human drug? of those who used antibiotics in pigs^2^ | | | |  |
| Yes | 0 (0.0) | 1 (1.2) | 0 (0.0) | 1 (0.6) |
| No | 56 (100.0) | 83 (98.8) | 21 (100.0) | 160 (99.4) |
| Which antibiotic have you used most frequently in pigs in the last 12 months (any route of administration) of those who used antibiotics in pigs^2^ | | | | |
| Penstrep | 3 (5.4) | 6 (7.1) | 3 (14.3) | 12 (7.5) |
| Medoxy-LA | 49 (87.5) | 66 (78.6) | 13 (61.9) | 128 (79.5) |
| Sulfa-strong/sulfabac | 0 (0.0) | 1 (1.2) | 2 (9.5) | 3 (1.9) |
| Oxytetracycline (Salep Mata) | 0 (0.0) | 1 (1.2) | 0 (0.0) | 1 (0.6) |
| Vet-Oxy LA | 4 (7.1) | 9 (10.7) | 0 (0.0) | 13 (8.1) |
| Oxytetracycline LA | 0 (0.0) | 0 (0.0) | 3 (14.3) | 3 (1.9) |
| Terramycin | 0 (0.0) | 1 (1.2) | 0 (0.0) | 1 (0.6) |
| Which antibiotics have you used in chickens in the last 12 months (any route of administration)^1, 3^ | | | |  |
| Penstrep | 13 (23.2) | 13 (15.3) | 1 (4.8) | 27 (16.7) |
| Medoxy-LA | 32 (57.1) | 41 (48.2) | 5 (23.8) | 78 (48.1) |
| Sulfa-strong/sulfabac | 3 (5.4) | 3 (3.5) | 2 (9.5) | 8 (4.9) |
| Oxytetracycline (Salep Mata) | 1 (1.8) | 2 (2.4) | 0 (0.0) | 3 (1.9) |
| Don't know | 0 (0.0) | 0 (0.0) | 0 (0.0) | 0 (0.0) |
| Don't use antibiotics in chickens | 16 (28.6) | 38 (44.7) | 13 (61.9) | 67 (41.4) |
| Vet-Oxy LA | 5 (8.9) | 6 (7.1) | 0 (0.0) | 11 (6.8) |
| Oxytetracycline LA | 0 (0.0) | 3 (3.5) | 1 (4.8) | 4 (2.5) |
| Tetra (human) | 0 (0.0) | 1 (1.2) | 1 (4.8) | 2 (1.2) |
| Terramycin | 0 (0.0) | 0 (0.0) | 1 (4.8) | 1 (0.6) |
| Tetrachlor (tablet) | 0 (0.0) | 0 (0.0) | 1 (4.8) | 1 (0.6) |
| Trimezyn (oral) | 0 (0.0) | 1 (1.2) | 0 (0.0) | 1 (0.6) |
| Colibact | 2 (3.6) | 0 (0.0) | 0 (0.0) | 2 (1.2) |
| Amoxicillin (human) | 1 (1.8) | 0 (0.0) | 1 (4.8) | 2 (1.2) |
| Entrocolin (oral) | 0 (0.0) | 0 (0.0) | 0 (0.0) | 0 (0.0) |
| Listed drug is not an antibiotic | 0 (0.0) | 1 (1.2) | 0 (0.0) | 1 (0.6) |
| What are the classes of drugs used in chickens in the last 12 months? (any route of administration) of those who used antibiotics in chickens^1, 3^ | | | | |
| Tetracycline | 35 (87.5) | 43 (91.5) | 7 (87.5) | 85 (89.5) |
| Penicillin | 14 (35.0) | 13 (27.7) | 2 (25.0) | 29 (30.5) |
| Aminoglycoside | 13 (32.5) | 13 (27.7) | 1 (12.5) | 27 (28.4) |
| Sulfonamide | 4 (10.0) | 4 (8.5) | 2 (25.0) | 10 (10.5) |
| Polypeptides | 0 (0.0) | 1 (2.1) | 0 (0.0) | 1 (1.1) |
| Was the antibiotics used in chickens in the last 12 months a human drug? of those who used antibiotics in chickens^3^ | | | | |
| Yes | 1 (2.5) | 1 (2.1) | 1 (12.5) | 3 (3.2) |
| No | 39 (97.5) | 46 (97.9) | 7 (87.5) | 92 (96.8) |
| Which antibiotic have you used most frequently in chickens in the last 12 months (any route of administration) of those who used antibiotics in chickens^3^ | | | | |
| Penstrep | 5 (12.5) | 5 (10.6) | 0 (0.0) | 10 (10.5) |
| Medoxy-LA | 30 (75.0) | 37 (78.7) | 5 (62.5) | 72 (75.8) |
| Sulfa-strong/sulfabac | 1 (2.5) | 0 (0.0) | 1 (12.5) | 2 (2.1) |
| Oxytetracycline (Salep Mata) | 0 (0.0) | 1 (2.1) | 0 (0.0) | 1 (1.1) |
| Depends | 0 (0.0) | 1 (2.1) | 0 (0.0) | 1 (1.1) |
| Vet-Oxy LA | 3 (7.5) | 2 (4.3) | 0 (0.0) | 5 (5.3) |
| Oxytetracyline LA | 0 (0.0) | 0 (0.0) | 1 (12.5) | 1 (1.1) |
| Tetrachlor (tablet) | 0 (0.0) | 0 (0.0) | 1 (12.5) | 1 (1.1) |
| Amoxicillin (human) | 1 (2.5) | 0 (0.0) | 0 (0.0) | 1 (1.1) |
| Not antibiotic | 0 (0.0) | 1 (2.1) | 0 (0.0) | 1 (1.1) |

^1^Multiple responses allowed

^2^Of those who reported using antibiotics in pigs (n = 161)

^3^Of those who reported using antibiotics in chickens (n = 95)

**Supplementary Table 10: Absolute and relative frequency of use of human and oral antibiotics by position and overall**

| Question | Veterinary Technicians  (n=56) | Livestock Technicians  (n=85) | Other Positions  (n=21) | Overall  (n=162) |
| --- | --- | --- | --- | --- |
| Given human antibiotics to animals |  |  |  |  |
| Yes | 9 (16.1) | 11 (12.9) | 5 (23.8) | 25 (15.4) |
| No | 47 (83.9) | 74 (87.1) | 16 (76.2) | 137 (84.6) |
| Given human antibiotics to which species^1, 2^ | |  |  |  |
| Local chicken | 5 (8.9) | 4 (4.8) | 5 (23.8) | 14 (8.7) |
| Broiler | 0 (0.0) | 0 (0.0) | 0 (0.0) | 0 (0.0) |
| Layer | 0 (0.0) | 0 (0.0) | 0 (0.0) | 0 (0.0) |
| Fighting cock | 2 (3.6) | 2 (2.4) | 2 (9.5) | 6 (3.7) |
| Pig | 3 (5.4) | 4 (4.8) | 2 (9.5) | 9 (5.6) |
| Cattle | 0 (0.0) | 4 (4.8) | 0 (0.0) | 4 (2.5) |
| Buffalo | 0 (0.0) | 0 (0.0) | 0 (0.0) | 0 (0.0) |
| Goat | 0 (0.0) | 2 (2.4) | 0 (0.0) | 2 (1.2) |
| Dog | 2 (3.6) | 2 (2.4) | 4 (19.0) | 8 (5.0) |
| Cat | 0 (0.0) | 0 (0.0) | 0 (0.0) | 0 (0.0) |
| Others | 0 (0.0) | 0 (0.0) | 0 (0.0) | 0 (0.0) |
| Observed human antibiotics given to animals in Timor-Leste | |  |  |  |
| Yes | 25 (44.6) | 20 (23.5) | 4 (19.0) | 49 (30.2) |
| No | 31 (55.4) | 65 (76.5) | 17 (81.0) | 113 (69.8) |
| Given antibiotics though water to animals |  |  |  |  |
| Yes | 9 (16.1) | 3 (3.5) | 3 (14.3) | 15 (9.3) |
| No | 47 (83.9) | 82 (96.5) | 18 (85.7) | 147 (90.7) |
| Don't know | 0 (0.0) | 0 (0.0) | 0 (0.0) | 0 (0.0) |
| Given antibiotics in water to which species^1,3^ | |  |  |  |
| Local chicken | 4 (44.4) | 0 (0.0) | 2 (66.7) | 6 (40.0) |
| Broiler | 0 (0.0) | 1 (33.3) | 0 (0.0) | 1 (6.7) |
| Layer | 2 (22.2) | 1 (33.3) | 0 (0.0) | 3 (20.0) |
| Fighting cock | 0 (0.0) | 0 (0.0) | 1 (33.3) | 1 (6.7) |
| Pig | 3 (33.3) | 1 (33.3) | 0 (0.0) | 4 (26.7) |
| Cattle | 2 (22.2) | 0 (0.0) | 1 (33.3) | 3 (20.0) |
| Horse | 1 (11.1) | 0 (0.0) | 0 (0.0) | 1 (6.7) |
| Goat | 0 (0.0) | 0 (0.0) | 1 (33.3) | 1 (6.7) |
| What antibiotic was given through water?^1,3^ | |  |  |  |
| Cola Floxa | 4 (44.4) | 0 (0.0) | 0 (0.0) | 4 (26.7) |
| Entrocolin | 2 (22.2) | 0 (0.0) | 0 (0.0) | 2 (13.3) |
| Koleridine | 2 (22.2) | 0 (0.0) | 0 (0.0) | 2 (13.3) |
| Vitachicks | 0 (0.0) | 2 (66.7) | 0 (0.0) | 2 (13.3) |
| Amoxitin | 1 (11.1) | 0 (0.0) | 0 (0.0) | 1 (6.7) |
| Interflox | 1 (11.1) | 0 (0.0) | 0 (0.0) | 1 (6.7) |
| Injectable oxytetracycline | 1 (11.1) | 0 (0.0) | 0 (0.0) | 1 (6.7) |
| Amoxicillin | 0 (0.0) | 0 (0.0) | 1 (33.3) | 1 (6.7) |
| Ampicillin | 0 (0.0) | 0 (0.0) | 1 (33.3) | 1 (6.7) |
| Penicillin | 1 (11.1) | 0 (0.0) | 0 (0.0) | 1 (6.7) |
| Sulphonamide powder | 1 (11.1) | 0 (0.0) | 0 (0.0) | 1 (6.7) |
| Cannot recall | 0 (0.0) | 1 (33.3) | 2 (66.7) | 3 (20.0) |
| What was the active ingredient? ^1,3^ |  |  |  |  |
| Enrofloxacin | 4 (44.4) | 0 (0.0) | 0 (0.0) | 4 (26.7) |
| Neomycin | 2 (22.2) | 0 (0.0) | 0 (0.0) | 2 (13.3) |
| Colistin | 3 (33.3) | 0 (0.0) | 0 (0.0) | 3 (20.0) |
| Amoxicillin | 3 (33.3) | 0 (0.0) | 1 (33.3) | 4 (26.7) |
| Penicillin | 0 (0.0) | 0 (0.0) | 1 (33.3) | 1 (6.7) |
| Oxytetracycline | 1 (11.1) | 0 (0.0) | 0 (0.0) | 1 (6.7) |
| Sulphonamide | 3 (33.3) | 0 (0.0) | 0 (0.0) | 3 (20.0) |
| Bacitracin | 1 (11.1) | 0 (0.0) | 0 (0.0) | 1 (6.7) |
| Cannot recall | 0 (0.0) | 2 (66.7) | 0 (0.0) | 2 (13.3) |
| What class of antibiotic? ^1,3^ |  |  |  |  |
| Fluorquinolone | (0.0) | (0.0) | (0.0) | (0.0) |
| Aminoglycoside | 4 (44.4) | 0 (0.0) | 0 (0.0) | 4 (26.7) |
| Polypeptide | 2 (22.2) | 0 (0.0) | 0 (0.0) | 2 (13.3) |
| Penicillin | 3 (33.3) | 2 (66.7) | 0 (0.0) | 5 (33.3) |
| Tetracycline | 4 (44.4) | 0 (0.0) | 1 (33.3) | 5 (33.3) |
| Sulphonamide | 3 (33.3) | 0 (0.0) | 0 (0.0) | 3 (20.0) |
| Cannot recall | 1 (11.1) | 0 (0.0) | 0 (0.0) | 1 (6.7) |
| Observed antibiotics given to animals through water in Timor-Leste | |  |  |  |
| Yes | 11 (19.6) | 9 (10.6) | 4 (19.0) | 24 (14.8) |
| No | 45 (80.4) | 76 (89.4) | 17 (81.0) | 138 (85.2) |
| Given antibiotics though feed to animals |  |  |  |  |
| Yes | 0 (0.0) | 3 (3.5) | 0 (0.0) | 3 (1.9) |
| No | 56 (100.0) | 81 (95.3) | 21 (100.0) | 158 (97.5) |
| Don't know | 0 (0.0) | 1 (1.2) | 0 (0.0) | 1 (0.6) |
| Observed antibiotics given to animals through feed in Timor-Leste | |  |  |  |
| Yes | 6 (10.7) | 4 (4.7) | 5 (23.8) | 15 (9.3) |
| No | 49 (87.5) | 81 (95.3) | 16 (76.2) | 146 (90.1) |
| Don't know | 1 (1.8) | 0 (0.0) | 0 (0.0) | 1 (0.6) |

^1^Multiple responses allowed

^2^Of those who had given human antibiotics to animals (n = 25)

^3^Of those who had given antibiotics to animals in water (n = 15)

**Supplementary Table 11: Absolute and relative frequency of antibiotic prescribing/administration practices by position and overall**

| Question | Veterinary Technicians  (n=56) | Livestock Technicians  (n=85) | Other Positions  (n=21) | Overall  (n=162) |
| --- | --- | --- | --- | --- |
| Check the expiry date of antibiotics before using |  |  |  |  |
| Always | 44 (78.6) | 69 (81.2) | 19 (90.5) | 132 (81.5) |
| Most of the time | 7 (12.5) | 8 (9.4) | 0 (0.0) | 15 (9.3) |
| Sometimes | 5 (8.9) | 7 (8.2) | 1 (4.8) | 13 (8.0) |
| Never | 0 (0.0) | 1 (1.2) | 1 (4.8) | 2 (1.2) |
| Ever given a lower antibiotic dose than on the label | |  |  |  |
| Yes | 27 (48.2) | 22 (25.9) | 3 (14.3) | 52 (32.1) |
| No | 28 (50.0) | 61 (71.8) | 18 (85.7) | 107 (66.0) |
| Don't know | 1 (1.8) | 2 (2.4) | 0 (0.0) | 3 (1.9) |
| Why yes ^1,2^ |  |  |  |  |
| Animal is very sick or weak | 16 (59.3) | 10 (45.5) | 2 (66.7) | 28 (53.8) |
| Inaccurate weight estimation | 4 (14.8) | 5 (22.7) | 1 (33.3) | 10 (19.2) |
| Lack of antibiotic supply | 2 (7.4) | 5 (22.7) | 2 (66.7) | 9 (17.3) |
| Others - give lower dose for disease prevention | 0 (0.0) | 1 (4.5) | 0 (0.0) | 1 (1.9) |
| Others - give half the dose because Timorese animals are different | 0 (0.0) | 1 (4.5) | 0 (0.0) | 1 (1.9) |
| Others - give lower dose because obliged to do so although it may not work | 1 (3.7) | 0 (0.0) | 0 (0.0) | 1 (1.9) |
| Others - give lower dose to insistent farmer | 1 (3.7) | 0 (0.0) | 0 (0.0) | 1 (1.9) |
| Others - give lower dose when used in combination with other veterinary medicines | 1 (3.7) | 0 (0.0) | 0 (0.0) | 1 (1.9) |
| Others - give lower dose to pregnant animals | 1 (3.7) | 0 (0.0) | 0 (0.0) | 1 (1.9) |
| Others - give lower dose for small wounds | 1 (3.7) | 0 (0.0) | 0 (0.0) | 1 (1.9) |
| Why no ^1,3^ |  |  |  |  |
| Need to follow instructions - on label | (0.0) | (0.0) | (0.0) | (0.0) |
| Underdosing is ineffective | 44 (157.1) | 69 (113.1) | 19 (105.6) | 132 (123.4) |
| Need to follow instructions - from other technicians | 7 (25.0) | 8 (13.1) | 0 (0.0) | 15 (14.0) |
| Give a dose according to estimated liveweight | 5 (17.9) | 7 (11.5) | 1 (5.6) | 13 (12.1) |
| Did not answer question - gave a higher dose in the past | 0 (0.0) | 1 (1.6) | 1 (5.6) | 2 (1.9) |
| Ever given a shorter duration of antibiotic treatment than on the label | |  |  |  |
| Yes | 22 (39.3) | 20 (23.5) | 6 (28.6) | 48 (29.6) |
| No | 34 (60.7) | 63 (74.1) | 15 (71.4) | 112 (69.1) |
| Don't know | 0 (0.0) | 2 (2.4) | 0 (0.0) | 2 (1.2) |
| Why yes^1,4^ |  |  |  |  |
| Animal has recovered | 10 (45.5) | 9 (45.0) | 2 (33.3) | 21 (43.8) |
| Lack of antibiotic supply | 3 (13.6) | 4 (20.0) | 4 (66.7) | 11 (22.9) |
| Farm in distant location and unable to continue treatment | 6 (27.3) | 3 (15.0) | 1 (16.7) | 10 (20.8) |
| Animal released by farmer and unable to continue treatment | 1 (4.5) | 2 (10.0) | 0 (0.0) | 3 (6.3) |
| Busy with work | 2 (9.1) | 0 (0.0) | 1 (16.7) | 3 (6.3) |
| Experiment with shorter dose | 1 (4.5) | 0 (0.0) | 0 (0.0) | 1 (2.1) |
| Worried that long duration of treatment could be deadly | 0 (0.0) | 1 (5.0) | 0 (0.0) | 1 (2.1) |
| Animal has been sold | 0 (0.0) | 1 (5.0) | 0 (0.0) | 1 (2.1) |
| Stop treatment because farmer is not providing good and sufficient feed as instructed to animal | 0 (0.0) | 1 (5.0) | 0 (0.0) | 1 (2.1) |
| Why no^1,5^ |  |  |  |  |
| Follow instructions - drug label instructions | 33 (97.1) | 55 (87.3) | 13 (86.7) | 101 (90.2) |
| Follow instructions - verbal instructions from other technicians | 0 (0.0) | 1 (1.6) | 2 (13.3) | 3 (2.7) |
| Follow instructions - non-specified | 1 (2.9) | 5 (7.9) | 0 (0.0) | 6 (5.4) |
| Treated for longer duration because of sick animal | 0 (0.0) | 3 (4.8) | 0 (0.0) | 3 (2.7) |
| Advise farmers to wait for a few days after giving antibiotics before slaughter/sale/eating | | |  |  |
| Always | 47 (83.9) | 59 (69.4) | 17 (81.0) | 123 (75.9) |
| Most of the time | 5 (8.9) | 10 (11.8) | 1 (4.8) | 16 (9.9) |
| Sometimes | 2 (3.6) | 12 (14.1) | 0 (0.0) | 14 (8.6) |
| Never | 2 (3.6) | 4 (4.7) | 3 (14.3) | 9 (5.6) |
| What else to you tell farmers before/after giving antibiotics (all that apply, not read out - may be additional categories)^1^ | | | | |
| Possibility animal might not improve | 27 (48.2) | 38 (44.7) | 11 (52.4) | 76 (46.9) |
| Ask farmer to contact if animal remains unwell | 15 (26.8) | 23 (27.1) | 9 (42.9) | 47 (29.0) |
| Ask farmer to contact to update on health status | 1 (1.8) | 3 (3.5) | 0 (0.0) | 4 (2.5) |
| Ask farmer to contact if animal dies | 1 (1.8) | 0 (0.0) | 0 (0.0) | 1 (0.6) |
| Implement good animal management - nutrition | 8 (14.3) | 16 (18.8) | 4 (19.0) | 28 (17.3) |
| Implement good animal management - housing | 2 (3.6) | 6 (7.1) | 2 (9.5) | 10 (6.2) |
| Implement good animal management - hygiene and sanitation | 5 (8.9) | 5 (5.9) | 2 (9.5) | 12 (7.4) |
| Implement good animal management - non-specific | 4 (7.1) | 11 (12.9) | 1 (4.8) | 16 (9.9) |
| Implement good animal management - vaccination | 0 (0.0) | 1 (1.2) | 0 (0.0) | 1 (0.6) |
| Isolate sick animal | 11 (19.6) | 12 (14.1) | 1 (4.8) | 24 (14.8) |
| Avoid eating dead animals | 11 (19.6) | 16 (18.8) | 3 (14.3) | 30 (18.5) |
| Bury dead animals | 1 (1.8) | 4 (4.7) | 0 (0.0) | 5 (3.1) |
| Provide instructions for eating dead animals | 3 (5.4) | 4 (4.7) | 0 (0.0) | 7 (4.3) |
| Impact of antimicrobial resistance | 0 (0.0) | 0 (0.0) | 0 (0.0) | 0 (0.0) |
| Antibiotics were not used according to the label | 0 (0.0) | 0 (0.0) | 0 (0.0) | 0 (0.0) |
| Storage conditions (if antibiotic given to the farmer) | 0 (0.0) | 0 (0.0) | 0 (0.0) | 0 (0.0) |
| Duration of treatment required | 4 (7.1) | 5 (5.9) | 4 (19.0) | 13 (8.0) |
| Adverse effects | 1 (1.8) | 2 (2.4) | 0 (0.0) | 3 (1.9) |
| Importance of diagnostic testing | 0 (0.0) | 0 (0.0) | 0 (0.0) | 0 (0.0) |
| Others | 1 (1.8) | 2 (2.4) | 0 (0.0) | 3 (1.9) |
| Nothing | 0 (0.0) | 3 (3.5) | 1 (4.8) | 4 (2.5) |
| I don't use antibiotics in animals | 0 (0.0) | 0 (0.0) | 0 (0.0) | 0 (0.0) |
| Record antibiotics use on "vijilansia" forms |  |  |  |  |
| Always | 52 (92.9) | 76 (89.4) | 18 (85.7) | 146 (90.1) |
| Most of the time | 3 (5.4) | 3 (3.5) | 2 (9.5) | 8 (4.9) |
| Sometimes | 1 (1.8) | 2 (2.4) | 0 (0.0) | 3 (1.9) |
| Never | 0 (0.0) | 4 (4.7) | 1 (4.8) | 5 (3.1) |
| Ever given a farmers antibiotics to inject animals themselves | |  |  |  |
| Yes | 22 (39.3) | 13 (15.3) | 3 (14.3) | 38 (23.5) |
| No | 34 (60.7) | 72 (84.7) | 18 (85.7) | 124 (76.5) |
| Ever advised farmers to give antibiotics without first examining the animals | | |  |  |
| Yes | 2 (3.6) | 1 (1.2) | 0 (0.0) | 3 (1.9) |
| No | 54 (96.4) | 84 (98.8) | 21 (100.0) | 159 (98.1) |
| Know of farmers who do not speak with livestock or veterinary technician before using antibiotics in animals | | | |  |
| Yes | 31 (55.4) | 41 (48.2) | 7 (33.3) | 79 (48.8) |
| No | 25 (44.6) | 44 (51.8) | 14 (66.7) | 83 (51.2) |
| If yes, do you know why they do this? ^1,6^ |  |  |  |  |
| Have access to antibiotics from Indonesia | 5 (8.9) | 5 (5.9) | 0 (0.0) | 10 (6.2) |
| Have access to antibiotics - not stated | 4 (7.1) | 14 (16.5) | 3 (14.3) | 21 (13.0) |
| Have access to antibiotics - agriculture shop | 5 (8.9) | 4 (4.7) | 0 (0.0) | 9 (5.6) |
| Poor access to veterinary services | 3 (5.4) | 6 (7.1) | 1 (4.8) | 10 (6.2) |
| Farmers are knowlegable - self-learning | 4 (7.1) | 2 (2.4) | 0 (0.0) | 6 (3.7) |
| Farmers are knowlegable - relatives are knowledgable | 0 (0.0) | 3 (3.5) | 1 (4.8) | 4 (2.5) |
| Farmers are knowlegable - received former training (e.g. APS) | 2 (3.6) | 4 (4.7) | 0 (0.0) | 6 (3.7) |
| Farmers are knowlegable - source not stated | 6 (10.7) | 3 (3.5) | 2 (9.5) | 11 (6.8) |
| Others (e.g. no trust of technician, worried about treatment cost) | 2 (3.6) | 0 (0.0) | 0 (0.0) | 2 (1.2) |

^1^Multiple responses allowed

^2^Of those who had given a lower dose of antibiotic (n = 52)

^3^Of those who had never given a lower dose of antibiotic (n = 107)

^4^Of those who had given a shorter duration of antibiotic (n = 48)

^5^Of those who had never given a shorter duration of antibiotic (n = 112)

^6^Of those who know of farmers who do not speak with livestock or veterinary technician before using antibiotics in animals (n = 79)
